# The Masked Polar Group Incorporation (MPGI) Strategy in Drug Design: Effects of Nitrogen Substitutions on Combretastatin and Isocombretastatin Tubulin Inhibitors

**DOI:** 10.3390/molecules24234319

**Published:** 2019-11-26

**Authors:** Myriam González, Younes Ellahioui, Raquel Álvarez, Laura Gallego-Yerga, Esther Caballero, Alba Vicente-Blázquez, Laura Ramudo, Miguel Marín Folgado, Cristina Sanz, Manuel Medarde, Rafael Pelaéz

**Affiliations:** 1 Laboratorio de Química Orgánica y Farmacéutica, Departamento de Ciencias Farmacéuticas, Universidad de Salamanca, Campus Miguel de Unamuno, E-37007 Salamanca, Spain; mygondi@usal.es (M.G.); youn@usal.es (Y.E.); raquelalvarez@usal.es (R.Á.); gallego@usal.es (L.G.-Y.); escab@usal.es (E.C.); avicenteblazquez@usal.es (A.V.-B.); id00681470@usal.es (M.M.F.); cristina.scuesta@usal.es (C.S.); medarde@usal.es (M.M.); 2 Instituto de Investigación Biomédica de Salamanca (IBSAL), Facultad de Farmacia, Universidad de Salamanca, Campus Miguel de Unamuno, E-37007 Salamanca, Spain; 3 Centro de Investigación de Enfermedades Tropicales de la Universidad de Salamanca (CIETUS), Facultad de Farmacia, Universidad de Salamanca, Campus Miguel de Unamuno, E-37007 Salamanca, Spain; 4 Departamento de Biología y Geología, Física y Química Inorgánica, ESCET, Universidad Rey Juan Carlos, Calle Tulipán s/n, Móstoles, E-28933 Madrid, Spain; 5 Laboratory of Cell Death and Cancer Therapy, Department of Molecular Biomedicine, Centro de Investigaciones Biológicas, Consejo Superior de Investigaciones Científicas (CSIC), E-28040 Madrid, Spain; 6 Departamento de Fisiología y Farmacología, Universidad de Salamanca, Campus Miguel de Unamuno, E-37007 Salamanca, Spain; ramudo@usal.es

**Keywords:** tubulin, colchicine-site, combretastatins, isocombretastatins, phenstatins, nitrogenated, masked polar group introduction, solubility, cytotoxicity, docking

## Abstract

Colchicine site ligands suffer from low aqueous solubility due to the highly hydrophobic nature of the binding site. A new strategy for increasing molecular polarity without exposing polar groups—termed masked polar group incorporation (MPGI)—was devised and applied to nitrogenated combretastatin analogues. Bulky *ortho* substituents to the pyridine nitrogen hinder it from the hydrophobic pocket while increasing molecular polarity. The resulting analogues show improved aqueous solubilities and highly potent antiproliferative activity against several cancer cell lines of different origin. The more potent compounds showed moderate tubulin polymerization inhibitory activity, arrested the cell cycle of treated cells at the G_2_/M phase, and subsequently caused apoptotic cell death represented by the cells gathered at the subG_0_/G_1_ population after 48 h of treatment. Annexin V/Propidium Iodide (PI) double-positive cells observed after 72 h confirmed the induction of apoptosis. Docking studies suggest binding at the colchicine site of tubulin in a similar way as combretastatin A4, with the polar groups masked by the vicinal substituents. These results validate the proposed strategy for the design of colchicine site ligands and open a new road to increasing the aqueous solubility of ligands binding in apolar environments.

## 1. Introduction

The microtubule filament system of eukaryotic cells is an important drug target for pesticides, antiparasitic and anticancer agents [1]. Cell microtubules show complex and highly regulated dynamic behavior essential for their functioning in cell shape maintenance, intracellular trafficking, cell motility and migration, and the formation of the mitotic spindle during cell division. The microtubule targeting agents (MTAs) bind to the main constituent of the microtubules, the αβ-tubulin dimers, in several different domains and alter their polymerization–depolymerization equilibria. They are often referred to as antimitotics, as their most patent effect is interference with the highly dynamic mitotic spindle, and depending on the effect they show on microtubule mass, they are often classified as microtubule stabilizing (MSA) or microtubule destabilizing agents (MDA). [2] The recent discovery that some types of MTAs, such as the colchicine-site binding drugs combretastatins, can shut down tumor neovasculature and behave as vascular disrupting agents (VDAs) has fostered an even greater interest in this class of drugs [3].

**Figure 1:**
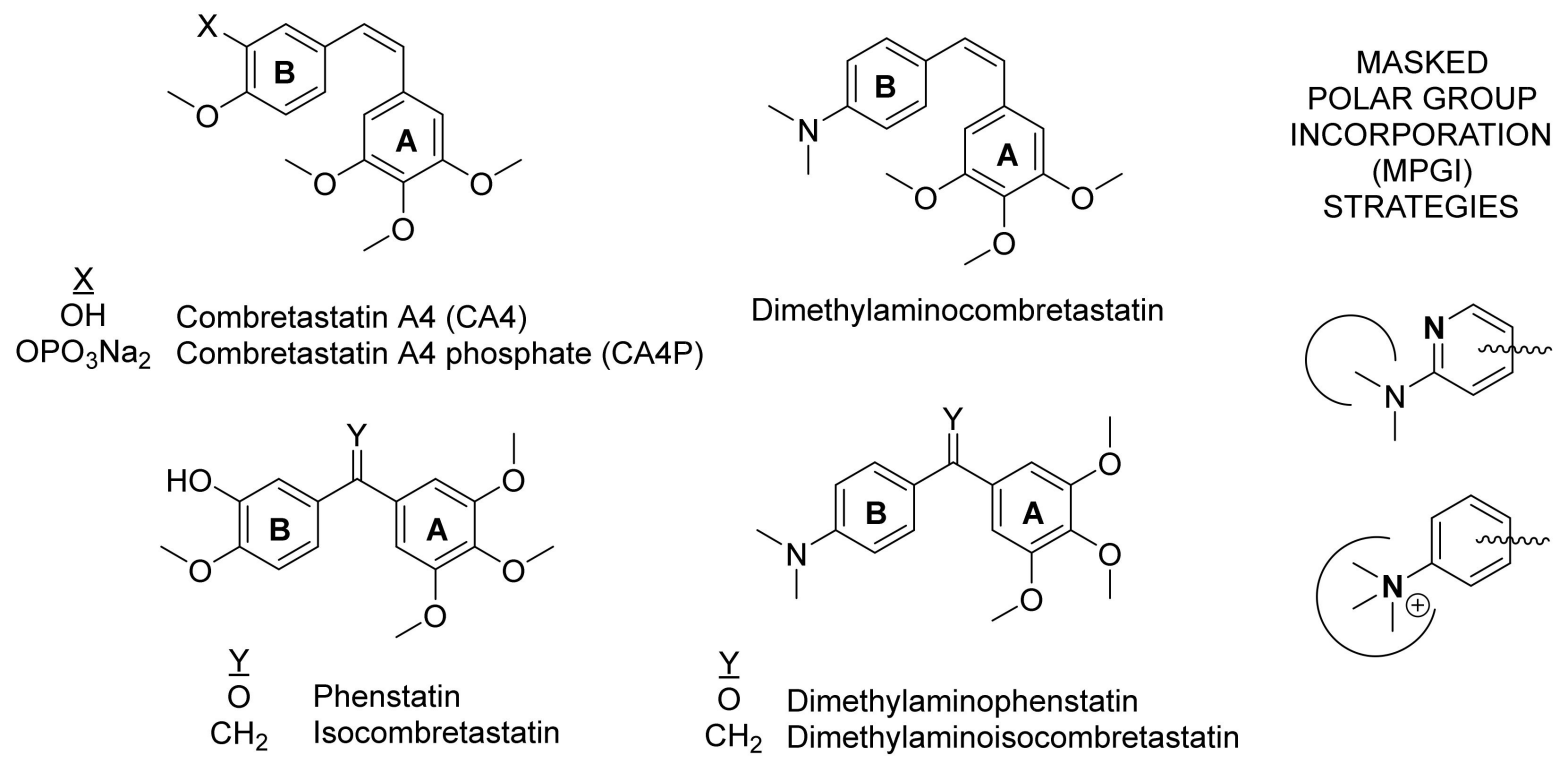
Structures of representative colchicine-site ligands and a summary of the masked polar group incorporation strategy followed in this work.

Combretastatin A4 (Figure 1) is the most prominent member of a family of natural product-derived MDAs, which is in clinical trials as a VDA [4]. Combretastatin A4 suffers from low water solubility, which has been circumvented by means of phosphate prodrug, and from isomerization of the *Z* stilbene to the less potent *E* isomer. Therefore, many combretastatin A4 modifications have been explored in order to provide new drugs with improved therapeutic profiles. The replacement of the olefinic bridge by small heterocycles [5] and its transformation into nonisomerizable one-atom bridges, such as the isocombretastatins [6,7], the phenstatins [8] and other derivatives have been amongst the most successful strategies aimed at increasing the configurational stability of combretastatins. The hydroxyl group used to anchor the phosphate in prodrug formation has recently been shown to represent an additional liability, as it suffers drug metabolism, which results in drug resistance [9]. Attempts to find alternatives to the 3-hydroxy-4-methoxyphenyl ring (B-ring) of combretastatin A4 have resulted in highly potent derivatives, such as indole [10,11,12,13] or naphthalene [13] analogues but with reduced water solubilities.

A dimethylaminophenyl ring can be thought of as an open analogue of an indole ring, and has been shown as an acceptable replacement for the B-ring of combretastatin A4 [14,15], but we have shown that it is not amenable to additional substitutions for solubility enhancement [16]. A frequent strategy applied to improve the solubility of drugs is the incorporation of polar groups, and to mask them as intramolecular hydrogen bonds [17]. However, this strategy can be difficult if the polar groups are placed in hydrophobic regions of the target, as it is the case with the colchicine site. Here, we propose a novel alternative strategy called masked polar group incorporation (MPGI), which implies the introduction of polar groups with vicinal bulky substituents in order to mask them from the outside, thus allowing binding at low polar binding sites while increasing the intrinsic water solubility. To this end, we have introduced nitrogen atoms on phenyl rings to form pyridines within the context of dimethylamino combretastatin and isocombretastatin analogues (Figure 1). A related strategy is the formation of ammonium salts with a polar core surrounded by hydrophobic alkyl groups. We have shown here that this new strategy is successful in achieving colchicine site ligands with improved intrinsic solubility profiles, while maintaining high antimitotic potency and in vitro activity associated with the inhibition of tubulin polymerization by binding at the colchicine site of tubulin, thus providing a proof of concept. These results guarantee further studies on these compounds, and provide a new strategy for the design of new colchicine site ligands with improved properties.

## 2. Results

### 2.1. Chemistry

#### 2.1.1. Chemical Synthesis

The phenstatin derivatives were prepared by two different methodologies. On one hand, compounds **1a**, **1b**, **1f**, and **1g** were obtained by one-step reactions between aromatic organolithium derivatives and sodium 3,4,5-trimethoxybenzoate. The starting organometallic compounds were synthesized by reaction between the bromo derivative (**A**, **B**, **F** or **G**) and *n*-butyllithium (*n*BuLi), whereas the sodium benzoate salt was prepared by treating 3,4,5-trimethoxybenzoic acid with sodium hydride. On the other hand, phenstatins **1c**, **1d**, **1e** and **1h** were alternatively prepared using a two-step synthesis. First, the organolithium compounds obtained from methoxybenzene derivatives **C’**, **D’**, or **E**’, afforded the intermediate alcohols by nucleophilic addition to aldehydes **C**, **D** or **H**. In a second step, oxidation of the diarylmethanol derivatives with pyridinium dichromate (PDC) gave the required diarylketones (**1c**, **1d**, **1e** and **1h**).

In order to increase the solubility of the compounds, ketone groups were replaced by oximes treating the phenstatins **1a**–**1h** with hydroxylamine hydrochloride and pyridine as catalyst in refluxing methanol to give **2a**–**2h**. In these reactions, a 1:1 *Z*/*E* isomer mixture that, in some cases, could be separated by column chromatography or by crystallization was obtained.

A second bridge modification was attempted by converting phenstatins **1a**–**1h** into isocombretastatins **3a**–**3h** through a Wittig reaction. The diarylketones were treated with triphenylphosphonium methylide, synthesized by reaction between methyltriphenylphosphonium iodide and *n*-butyllithium in dry tetrahydrofuran (THF).

To explore the effect of the double bond at the bridge of the diaryl compounds, the ethenylidene group was converted into ethyl group by reduction of isocombretastatins **3d**, **3e**, and **3f** under hydrogen atmosphere using Pd/C as catalyst to get **4d**, **4e**, and **4f.**

The importance of the double bond arrangement in these systems was assessed by comparing the properties of the synthesized isocombretastatins with their isomeric combretastatins analogues. They were accessed via Wittig reaction by the nucleophilic addition of triphenyl(3,4,5-trimethoxybenzyl)phosphonium iodide to the aldehydes **A’’**–**I’’** to obtain combretastatins **8a**–**8i.**

Finally, another strategy to increase the water solubility of the synthesized compounds involved the preparation of quaternary ammonium salts for some of the derivatives having dialkylamino groups at the B ring. For that purpose, phenstatins **1a, 1d,** and **1e**; oxime **2a**; isocombretastatins **3a** and **3d;** and combretastatins **8a, 8b,** and **8i** were treated with iodomethane in refluxing acetone to obtain the corresponding ammonium iodide derivatives.

**Scheme 1:**
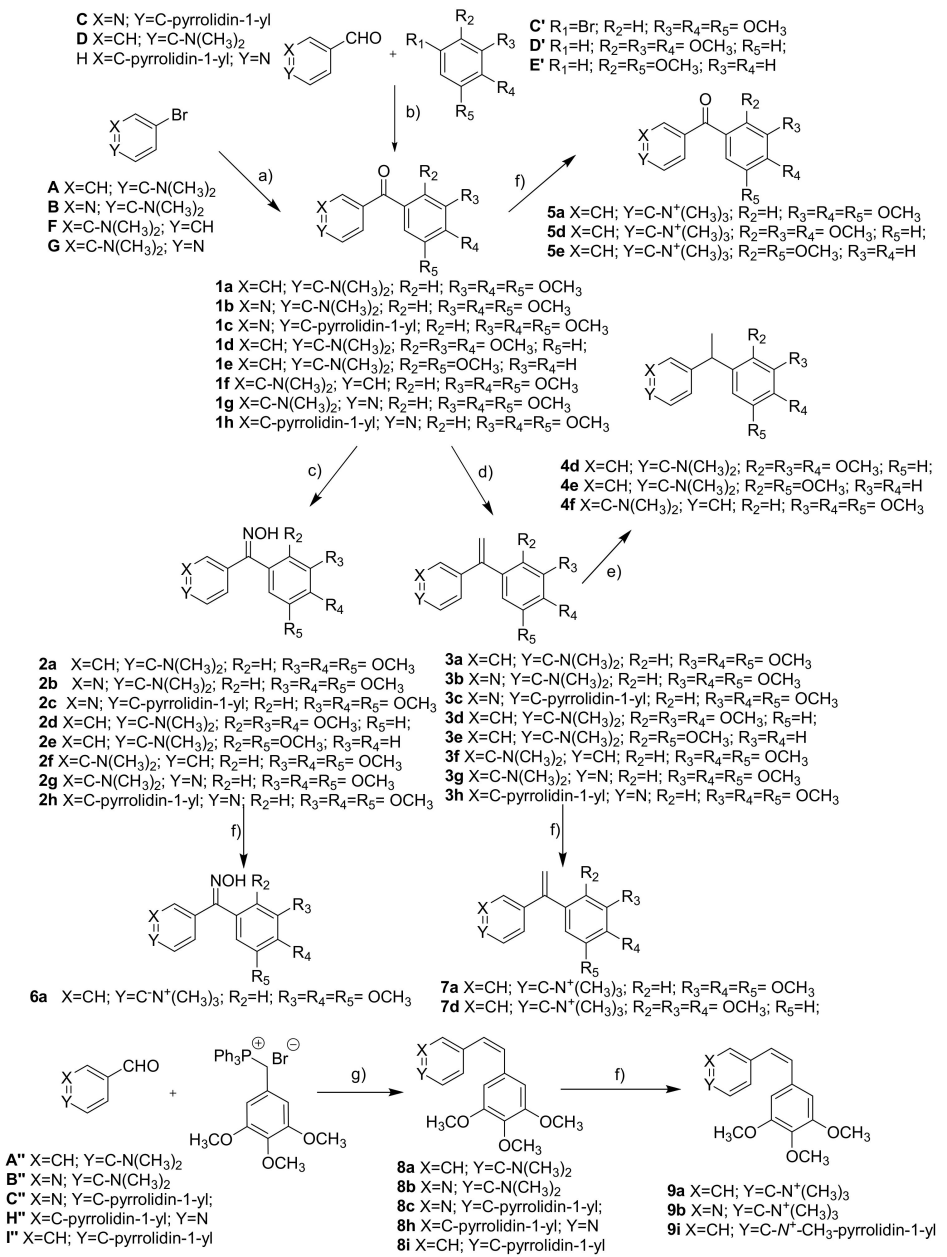
Synthesis of compounds **1a**–**9i**. (**a**) 1. *n*BuLi, THF, −78 °C; 2. 3,4,5-trimethoxybenzoic acid, NaH, 0 °C—rt; (**b**) 1. *n*BuLi, THF, −78 °C; 2. ArCHO, THF; 3. PDC, HSO_4_ Bu_4_N; (**c**) NH_2_OH·HCl, pyridine, MeOH, reflux; (**d**) Ph_3_PMeI, *n*BuLi, THF, −40 °C; (**e**) H_2_, Pd/C, EtOH, rt; (**f**) MeI, acetone, reflux; (**g**) *n*BuLi, THF, −78 °C.

The whole synthetic approach, which is detailed in Scheme 1, succeeded in obtaining a set of 41 diaryl compounds with several methoxy patterns at A ring, different nitrogen functional groups at B ring and modifications at the bridge to establish structure-activity relationship studies.

#### 2.1.2. Solubility

One of the expected effects of introducing nitrogen substitutions on combretastatin and isocombretastatin analogues, is an increase in aqueous solubility. Solubility was spectrophotometrically determined in phosphate buffer at pH 7. UV absorbance of soluble compounds was measured after shaking and microfiltration. Values are displayed in Table 1, showing a significant improvement of aqueous solubility in comparison to CA-4 (1 µg/mL): 10- to 150-fold in compounds with dimethylaminophenyl moieties, and 10- to 583-fold in compounds with a pyridine moiety. In addition, aqueous solubilities of the corresponding ammonium salts are over 1 mg/mL.

### 2.2. Biology

#### 2.2.1. Cell Viability

The in vitro antiproliferative activity of the synthesized compounds was evaluated by a colorimetric reaction, using the MTT reagent. They were tested against three different human tumor cell lines: HeLa cervix epithelioid carcinoma, HT-29 colon adenocarcinoma, and MCF-7 breast adenocarcinoma. The compounds were initially tested at 1 µM, and for those inhibiting cell proliferation at least by a 50%, the half maximal inhibitory concentration (IC_50_) values were calculated. *p-*Dimethylaminophenyl derivatives **1a**, **2a**, **3a** and **8a** were used for comparison between phenyl and pyridine analogues. Moving the alkylamino substituent from a *para* to a *meta* position with respect to the bridge results in potency reduction, except for the phenstatin analogue **1f**. However, the introduction of a nitrogen atom to form 2-dimethylaminopyridine derivatives entails a loss of activity, both for phenstatin analogues (**1b**, **2b**), while for isocombretastatins, it only involves a 2–3 times potency decrease (**3b**). For combretastatins, it implies an improvement in the cytotoxicity values down to tenths of nanomolar (**8b**). 2-Pyrrolidin-1-yl pyridine moieties resulted in highly potent antiproliferative phenstatins (i.e., compound **1h**). On the other hand, the best 2-dimethylaminopyridine was combretastatin **8b**. All ammonium salts derivatives were not cytotoxic at micromolar concentrations.

In order to test whether these compounds are substrates of multi-drug resistance (MDR) glycoproteins, we have adopted a pharmacological approach and measured the IC_50_ values against HT-29 in the presence of 10 µM verapamil, a known nonselective P-gP1/MDR1 inhibitor. As observed in Table 1, there are no significant changes in the IC_50_ values for most compounds, except for combretastatins **8a** and **8i.**

#### 2.2.2. Tubulin Polymerization Inhibition

The effects of cytotoxic compounds on in vitro tubulin assembly were studied at 20 µM concentration, and the IC_50_ values were determined for the more potent ones (**1h**, **3b** and **8b**), proving that tubulin is indeed the putative target of these compounds.

#### 2.2.3. Effects on the Cell Cycle

**Figure 2:**
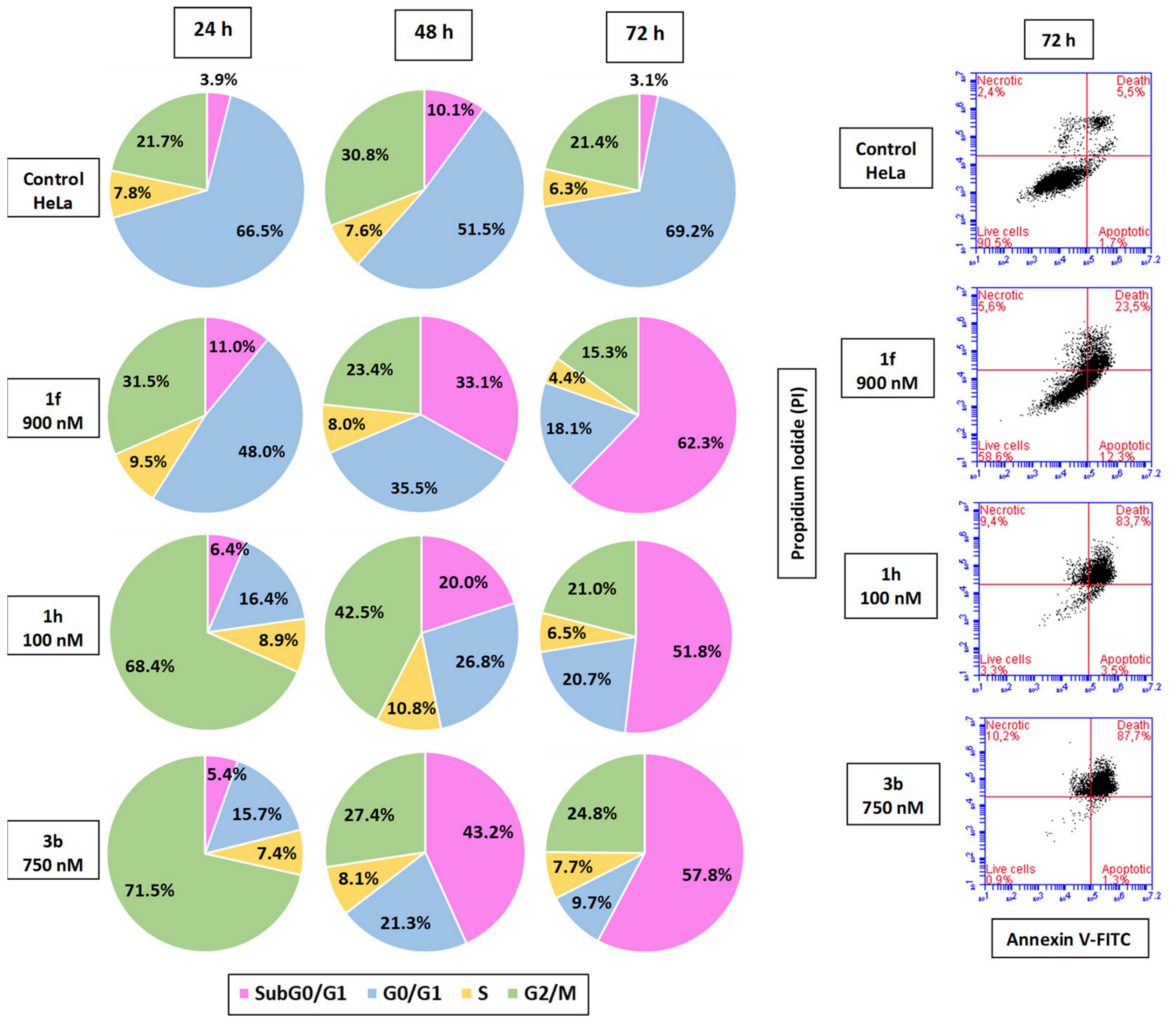
Annexin V-FITC and propidium iodide (PI) profiles, and pie charts of the different cell populations of the cell cycle of HeLa cells after 24, 48, and 72 h after treatment with the drugs at the indicated doses.

The effect of the most cytotoxic compounds (**1f**, **1h** and **3b**) on the cell cycle profiles of HeLa cells were studied at different time points (24, 48, and 72 h) by flow cytometry, measuring the relative DNA content. Compounds **1h** and **3b** significantly arrested HeLa cells at the G_2_/M phase after a 24-h incubation (68–71%, Figure 2, followed by an increase of the cell population at the subG_0_/G_1_ region, suggesting that cell death occurs via apoptosis. In order to assess the cell death mechanism, non-fixed cells were double stained with Annexin V-FITC and propidium iodide (PI), showing that cell death is mainly triggered by apoptosis, which is in agreement with the previous observations (Figure 2).

#### 2.2.4. Effect on Rat Primary Pancreatic Cells

The toxicity of the compounds against rat primary pancreatic cells was evaluated by the MTT protocol (Table 2) [18]. The compounds showed lower toxicity than doxorubicin, acetyl-digoxin, and etoposide used as positive controls, even at concentrations 5- to 10-fold higher. Sodium taurocholate (NaTc) was also used at high concentrations as a model of acute pancreatitis in vitro.

### 2.3. Molecular Modelling

Docking studies were performed with the proposed ligand molecules into the colchicine site of tubulin, using three programs with different scoring functions. The flexibility of the protein was accounted for by employing different tubulin structures: from X-ray structures of 50 complexes with different ligands in the colchicine site, and from selected representative snapshots of molecular dynamic simulations. For pose selection, a consensus score strategy was followed, therefore selecting those poses that are best scored by a combination of the three programs. In most cases, the ligands bind within the colchicine site in a similar way as combretastatin A4, with their trimethoxyphenyl rings close to the trimetoxyphenyl ring of combretastatin A4, and the nitrogenated phenyl rings placed in the 3-hydroxy-4-methoxyphenyl ring of combretastatin A4 pocket. Ammonium salts bind with a much lower affinity than their neutral analogues, and pyridines were bound in a very similar way as their phenyl counterparts, with the pyridine nitrogen hidden from the molecular surface by the bulky substituents, which interact favorably with the hydrophobic walls of the site.

## 3. Discussion

Combretastin A4 is arguably the most successful representative so far of the family of the colchicine site ligands [4,5]. However, the 3-hydroxy group of combretastatin A4 that provides an anchor for phosphate prodrug formulation is used by tumor cells for conjugation and development of resistance [9,19]. The replacement of the 3-hydroxy-4-methoxyphenyl ring of combretastatins, isocombretastatins and phenstatins by indoles [11,12,13,14,20,21], naphthalenes [13], and 4-dimethylaminophenyl rings [15,16,22], results in potent tubulin inhibitory and cytotoxic compounds, but require further elaboration in order to achieve improved water solubility [10,23]. An alternative to prodrug formation is the introduction of polar functions, which has been attempted by replacing the phenyl rings of combretastatins, isocombretastatins, and related compounds, with pyridine rings, yielding better results as A than as B rings [15,22,24,25,26]. This likely reflects the more hydrophobic nature of the pocket, and the energy penalty associated with the desolvation of these polar groups. Here, we propose a new strategy analogous to the intramolecular hydrogen bond concept, consisting on masquerading the hydrogen bond donor and the acceptor pairs in apolar environments by means of mutual interaction, which renders the molecules more hydrophobic. We propose the introduction of polar moieties on the structure of combretastatin analogues, such as the previously attempted pyridines, but masking them in the apolar environment by means of substituents, which hide them from the molecular surface: a strategy we have called masked polar group incorporation (MPGI). For the masking group we have selected alkylamino groups that have been previously shown to interact favorably with zone B, such as the dimethylamino indicated above. Earlier attempts of introducing pyridines with substituents such as 2-methoxypyridines would require the freezing of the methoxy group rotation for the masking of the pyridine nitrogen, and this might cause the observed reduction in potency [22].

With the aim of testing the aforementioned proposal in different scaffold contexts which have been shown to result in different structure–activity relationships [7,10,11,23,27], we have synthesized a new family of combretastatins (**8**), phenstatins (**1**), isocombretastatins (**3**), 1,1-diarylethanes (**4**), and diaryloxime derivatives (**2**) with a pyridine B–ring *ortho* substituted with dialkylamino groups and their phenyl counterparts for the sake of comparison. As a further extension of the strategy, ammonium salts of the dialkylamino substituents have been prepared (**5–7**, **9**), as they have polar (charged) groups surrounded by a hydrophobic cap. The synthetic strategies applied have been extensively described for the indicated compound classes. The combretastatins have been prepared by Wittig olefination reactions, the benzophenones by additions of organolithium aryls to carbonyls, and the oximes and isocombretastatins (1,1-diarylethenes) by the reaction of the corresponding benzophenones with hydroxylamine, and by Wittig olefination, respectively. Catalytic hydrogenation of the isocombretastatins produced the corresponding 1,1-diarylethanes in good yields. The ammonium salts were prepared by alkylation of the corresponding amines with methyl iodide.

The thermodynamic solubility of the synthesized compounds has been measured by the shaking flash methodology. All the compounds measured showed higher aqueous solubilities than combretastatin A4 (1 µg/mL). With respect to the bridge between the aromatic rings, the hydroxylamine derivatives showed the higher solubility, followed by the olefins, and the ketones were the least soluble. The high solubility of the oximes could be attributed to the polar nature of the bridge, but in previous studies related oximes showed the lowest solubilities [10,16]. Within the oximes, the 3-dimethylaminopyridine (**2g**) and 3-dimethylaminophenyl (**2f**) analogues showed a notable increase in solubility with respect to the 4-dimethylamino analogue **2a**, with **2g** in the 0.6 mg/mL range. This great improvement is probably due to the symmetry reduction [28], combined with an increased polarity for **2g**. All ammonium salts showed huge solubility improvements, with values in the µM range in all cases.

The antiproliferative activity of the compounds against three human tumor cell lines of different origins (i.e., HeLa cervix epithelioid carcinoma, HT-29 colon adenocarcinoma and MCF-7 breast adenocarcinoma) was evaluated by the MTT method. Sixteen out of the 40 tested compounds showed micromolar or less proliferation inhibitory potency. This ratio is improved to more than 50% (16 out of 31) if the ammonium salts are not considered, as they are all inactive. This high success rate is the result of a combination of favorable scaffolds and substituents, and it is similar to those of previous reports on related series [7,10,13,16,23]. Of the three cell lines, HeLa and MCF-7 showed similar sensitivities, while HT-29 cells were less susceptible. We have previously observed the same trend for related compounds acting at the colchicine site of tubulin [10,23]. For this pyridine series, we observed a different potency trend when we compare the 4-amino-3-pyridine and the 3-amino-4-pyridine derivatives. For the first ones, the most potent compounds are the combretastatin and the isocombretastatins, whereas for the second ones, the more potent analogues are the phenstatins. This different behavior is probably a reflection of their different shape, combined with the different geometries imposed by different bridges. We have previously shown that the potency ordering isocombretastatins similar to oximes higher than benzophenones could be explained by the higher conjugation of the ketones with the aromatic rings, which imposes a more planar disposition than those required for high affinity colchicine site binding [10]. For the 3-amino-4-pyridine series, more planar structures are required in order to avoid the bumping of the amino substituents with tubulin out the plane of the aromatic ring, an effect which we have previously shown to impact the potency of 3-substituted indolecombretastatins and dimethylaminophenyl phenstatins [16,23]. This different geometrical disposition of the amino substituents, and the different adaptability of their corresponding pockets in the two series, can also account for the preference of bulkier substituents at position 3, such as the pyrrolidine rather than the smaller dimethylamino. The opposite trend was observed for position 4. The 3,4,5-trimethoxyphenyl ring is strongly preferred over 2,3,4-trimethoxyphenyl, and 2,5-dimethoxyphenyl rings, which have also been found in highly potent colchicine site ligands [20]. The phenyl to pyridine substitution is favorable for the 4-dimethylamino combretastatins (i.e., compare **8a** and **8b**), and results in the most potent 3-pyrrolidin-1-yl phenstatin (**1h**). The opposite is observed for the 4-dimethylaminophenyl phenstatins **1a** and **1b**. The highly polar ammonium salts showed IC_50_ values higher than micromolar, and are considered inactive. The remarkable levels of solubility achieved for the quaternary ammonium salts came unfortunately at the expenses of biological activity.

Acquired resistance to antitumor drugs is one of the more important problems of chemotherapy, also affecting tubulin-targeting drugs [21]. In order to ascertain the impact of multidrug resistance phenotypes on the action of the compounds, we have measured the cell proliferation inhibitory activity of HT-29 cells in the presence of verapamil, a known P-glycoprotein 1/multidrug resistance protein 1 (MDR1) inhibitor at a concentration of 10 µM, not affecting cell proliferation [29]. Roughly one-third of the active compounds in the screen (**3b–c**, **3h**, **8a**, **8i**) showed an improvement bigger than 2-fold after verapamil treatment, thus suggesting that they might be MDR1 substrates. Except for the combretastatin analogues **8a** and **8i**, the affected drugs are all pyridine drugs. The most potent compounds of the series (i.e., combretastatin **8b** and ketone **1h**) seem not to be MDR1 substrates.

In order to confirm the tubulin inhibitory mechanism of action of the compounds, we have studied their effect on the in vitro assembly of bovine brain tubulin. Most of the compounds showed modest inhibition of tubulin polymerization at concentrations of 20 µM, but the most cytotoxic compounds showed moderate to good inhibitory potencies, with combretastatin **8b** and ketone **1h** having the lowest IC_50_ values of 5.1 and 9.1 µM, comparable to those of combretastatin A4 (1–4 µM) or reference compound **8a** (3.4 µM) [16]. The pyridine-containing compounds have higher IC_50_ values in TPI than their phenyl matched pairs (e.g., compare **8a** and **8b**), but they still show lower IC_50_ values in cytotoxicity. This lack of correlation between tubulin inhibition and cytotoxicity has previously been observed for series with different scaffolds and binding sites, and attributed to the accepted theory that the antiproliferative effects are due to interference with tubulin dynamics, and not with their effect on total polymer mass [1,20]. In any case, the tubulin inhibitory mechanism for the most potent compounds **8b** and ketone **1h** is clearly established.

We have studied the effect of **1f**, **1h,** and **3b** on the cell cycle of HeLa cells at different time points (24, 48 and 72 h) after drug treatment (Figure 2). After 24 h, a significant arrest of about 70% at the G_2_/M phase is observed for **1h** and **3b,** and a more modest one for **1f**. After 48 h, the cells are experiencing apoptosis as indicated by subG_0_/G_1_ fractions, especially for compound **3b** (45%). At a later time point of 72 h, the subG_0_/G_1_ fractions represent more than 50% of the cells for the three compounds. These results suggest that the compounds arrest the cells at the G_2_/M phase to different extents, and that this arrest leads to cell death. We have further characterized the cell death by means of flow cytometry using Annexin V/PI. PI is excluded from live cells, and only stains apoptotic or necrotic cells with altered membrane permeability. Apoptotic cells are Annexin V positive, as they have phosphatidylserine exposed on the surface as a result of its translocation from the inner cytoplasmic membrane from the beginning of apoptosis. The combined stain allows therefore to establish whether cells are viable (PI-/AnV-), early apoptotic (PI-/AnV+), or late apoptotic or secondary necrotic (PI+/AnV+). At 72 h, the results are consistent with the cell cycle profiles, with cells already in a PI+/AnV+ state. These results suggest that after an arrest at the G_2_/M phase cells suffer apoptosis.

The toxicity of the more antiproliferative compounds **1f**, **1h**, **3b** and **8b** against primary pancreatic cultured cells was measured and compared with doxorubicin (a DNA intercalator), etoposide (a topoisomerase II poison), acetyl-digoxin (a cardiotonic steroid), and sodium taurocholate (an in vitro model of acute pancreatitis). Compounds **1f**, **1h**, **3b** and **8b** showed very low toxicity against primary pancreatic cultured cells (Table 2).

**Figure 3:**
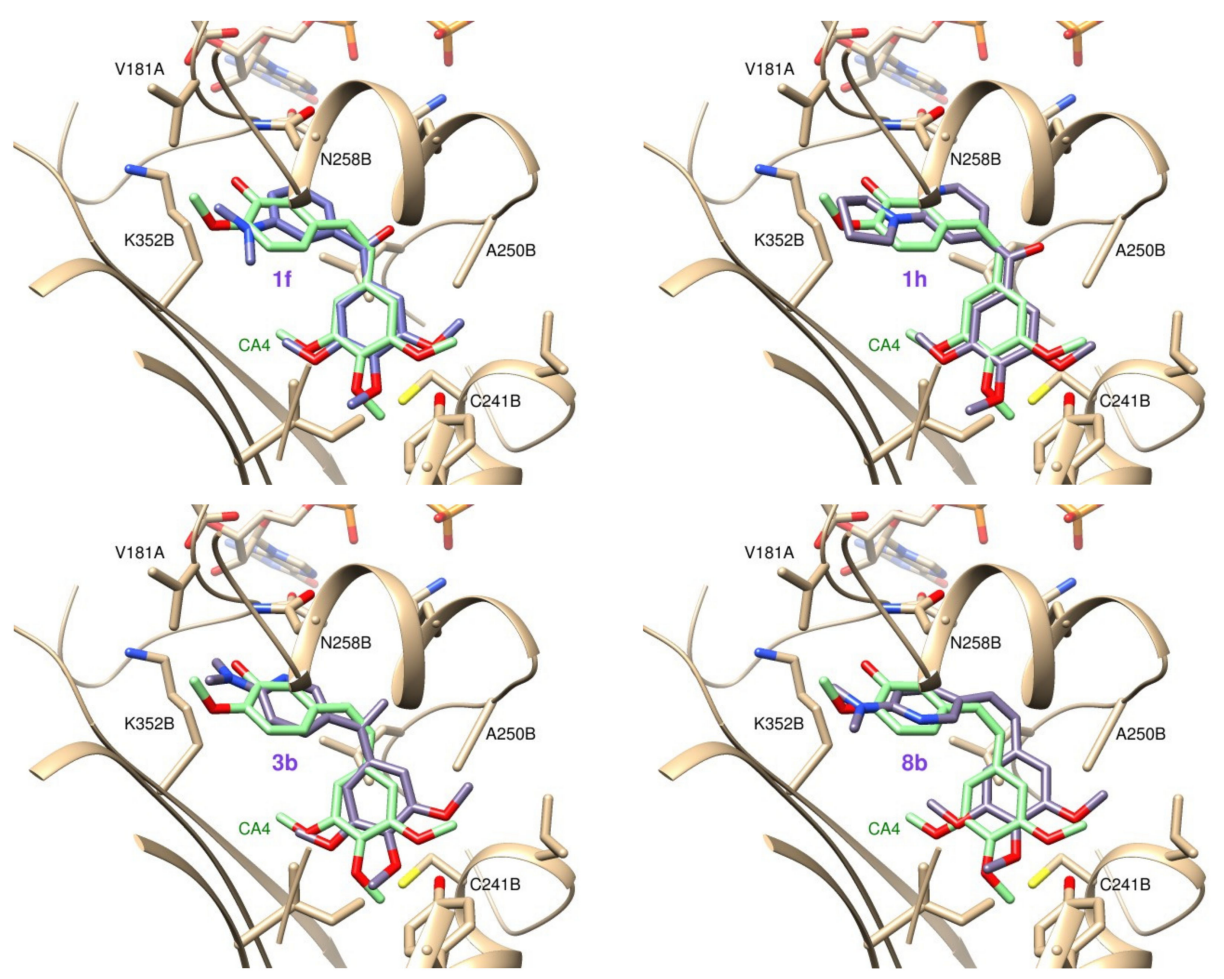
Docking poses for compounds **1f**, **1h**, **3b** and **8b** superimposed with the X-ray pose of CA-4 (light green).

We have performed docking studies in order to determine the binding modes of the synthesized compounds. The protein flexibility was accounted for by using 50 X-ray structures of tubulin in complex with different ligands at the colchicine site [20] and five additional ones from a molecular dynamics simulation, and the ligand flexibility was considered explicitly [21]. The binding poses from three docking programs (PLANTS [30], AutoDock [31], and DOCK [32]) with substantially different scoring functions and for each docked ligand, we selected the binding pose that scored higher for the three programs (Supplementary Table S1). Using this consensus score all the tested ligands docked at the zone A with the methoxylated ring and the other ring at zone B (Figure 3). In all cases, the pyridine ring docks into a hydrophobic region of the protein that contacts the hydrophobic substituent nearby and with the pyridine nitrogen in a nonpolar environment, in good agreement with the MPGI strategy.

## 4. Materials and Methods

Reagents were used as purchased without further purification. Solvents (THF, DMF, CH_2_Cl_2_) were dried and freshly distilled before use according to procedures reported in the literature. Chromatographic separations were performed on silica gel columns by flash (Kieselgel 40, 0.040–0.063; Merck). TLC was performed on precoated silica gel polyester plates (0.25 mm thickness, with UV 254 fluorescent indicator). Melting points were determined on a Buchi 510 apparatus and are uncorrected. ^1^H NMR and ^13^C NMR spectra were recorded on a Bruker AC200 spectrometer at 200/50 MHz, on a Bruker SY spectrometer at 400/100 MHz or on a Varian Mercury 400/100 MHz, using CDCl_3_ as solvent (unless otherwise stated). Chemical shifts (δ) are given in ppm downfield from tetramethylsilane, and coupling constants (*J* values) are in Hertz. Electrospray-ionisation (ESI) high-resolution mass spectra (HRMS) were obtained on a VG-TS250 apparatus (70 eV). A Helios Alfa UV-320 from Thermo-Spectronic was used for UV spectra and turbidimetric measurements. IR spectra were acquired in a FTIR Nicolet Impact 410 equipment (film, unless otherwise stated) and the absorptions are expressed in cm^−1^. The aqueous solubility of the compound was determined in a Helios Alfa Spectrophotometer.

### 4.1. Synthesis

#### 4.1.1. General Procedure to Obtain Diarylketones by One-step Synthesis (**1a**, **1b**, **1f**, **1g**)

1.5 mmol per mmol of 1.6 M *n*BuLi in hexane was added to a stirred 0.2 M solution of the corresponding bromo derivative in dry THF at −78 °C under an inert atmosphere. After 1 h, the colored solution was added to a 0.2 M suspension of the corresponding sodium benzoate in dry THF at 0 °C, previously formed by adding 3 mmol per mmol of sodium hydride over 2 mmol per mmol of the benzoic acid. After 24 h at room temperature, the reaction was poured onto ammonium chloride, extracted with ethyl acetate and the organic layers were dried over anhydrous sodium sulfate, filtered and dried under vacuum. The reaction crude was purified by column chromatography.

*(4-(Dimethylamino)phenyl)(3,4,5-trimethoxyphenyl)methanone* (**1a**) [16]. 3.50 g (17.5 mmol) of *p-*bromodimethylaniline in THF treated with 16.4 mL (26.2 mmol) of *n*BuLi 1.6 M in hexane at −78 °C were added to 35.0 mmol of 3,4,5-trimethoxybenzoate in dry THF to obtain 4.22 g of reaction crude. After flash chromatography Hex/EtOAc 1:1, 2.50 g (7.93 mmol, 46%) of compound **1a** were isolated. ^1^H-NMR δ: 3.06 (6H, s); 3.85 (6H, s); 3.90 (3H, s); 6.66 (2H, d, 8.6); 6.98 (2H, s); 7.80 (2H, d, 8.6). ^13^C-NMR δ: 40.1 (2) (CH_3_); 56.3 (2) (CH_3_); 61.0 (CH_3_); 107.1 (2) (CH); 110.6 (2) (CH); 124.8 (C); 132.7 (2) (CH); 134.5 (C); 140.8 (C); 152.8 (2) (C); 153.3 (C); 194.4 (C). IR (film): 1681; 1583; 1411; 1328; 1121 cm^−1^. Mp (Diisopropylether): 107–109 °C. HRMS: calculated for C_18_H_21_NO_4_ [M + Na]^+^: 338.1368; found: 338.1363.

*(6-(Dimethylamino)pyridin-3-yl)(3,4,5-trimethoxyphenyl)methanone* (**1b**). 627 mg (3.12 mmol) of 5-bromo-*N,N*-dimethylpiridin-2-amine in THF treated with 1.9 mL (3.1 mmol) of *n*BuLi 1.6 M in hexane at −78 °C were added to 6.22 mmol of 3,4,5-trimethoxybenzoate in dry THF to obtain 1.25 g of reaction crude. After flash chromatography Hex/EtOAc 9:1, 833 mg (2.63 mmol, 84%) of compound **1b** were isolated. ^1^H-NMR δ: 3.20 (6H, s); 3.88 (6H, s); 3.91 (3H, s); 6.56 (1H, d, 9,2); 6.99 (2H, s); 8.01 (1H, dd, 9.2, 2.4); 8.63 (1H, d, 2.4). ^13^C-NMR δ: 38.2 (2) (CH_3_); 56.3 (2) (CH_3_); 60.9 (CH_3_); 105.1 (CH); 107.0 (2) (CH); 121.2 (C); 133.7 (C); 138.8 (CH); 143.4 (C); 152.4 (CH); 152.8 (2) (C); 160.4 (C); 187.6 (C). IR (KBr): 1686; 1608; 1584; 1508; 1403; 1337; 1132 cm^−1^. Mp (Diisopropylether): 119–120 °C. HRMS: calculated for C_17_H_21_N_2_O_4_ [M + H]^+^: 317.1501; found: 317.1507.

*(3-(Dimethylamino)phenyl)(3,4,5-trimethoxyphenyl)methanone* (**1f**). 2.78 g (13.9 mmol) of 3-bromodimethylaniline in THF treated with 9.5 mL (15 mmol) of *n*BuLi 1.6 M in hexane at −78 °C were added to 20.8 mmol of 3,4,5-trimethoxybenzoate in dry THF to obtain 3.27 g of reaction crude. After flash chromatography Hex/EtOAc 7:3, 3.27 g (10.4 mmol, 75%) of compound **1f** were isolated. ^1^H-NMR δ: 2.89 (6H, s); 3.76 (6H, s); 3.83 (3H, s); 6.82 (1H, dd, 8.0, 2.8); 6.95 (1H, bd, 8.0); 6.99 (2H, s); 7.04 (1H, bs); 7.29 (1H, t, 8.0). ^13^C-NMR δ: 40.5 (2) (CH_3_); 56.3 (2) (CH_3_); 61.0 (CH_3_); 107.8 (2) (CH); 113.2 (CH); 116.2 (CH); 118.3 (CH); 128.8 (CH); 133.0 (C); 138.5 (C); 141.8 (C); 150.4 (C); 152.8 (2) (C); 196.4 (C). IR (KBr): 1650; 1589; 1412; 1334; 1230; 1124 cm^−1^. Mp (methanol): 77–78 °C. HRMS: calculated for C_18_H_21_NO_4_ [M + Na]^+^: 338.1368; found: 338.1361.

*(2-(Dimethylamino)pyridin-4-yl)(3,4,5-trimethoxyphenyl)methanone* (**1g**). 713 mg (3.54 mmol) of 4-bromo-*N,N*-dimethylpyridin-2-amine in THF treated with 2.9 mL (4.6 mmol) of *n*BuLi 1.6 M in hexane at −78 °C were added to 7.07 mmol of 3,4,5-trimethoxybenzoate in dry THF to obtain 874 mg of reaction crude. After flash chromatography Hex/EtOAc 7:3, 282 mg (0.90 mmol, 26%) of compound **1g** were isolated. ^1^H-NMR δ: 3.13 (6H, s); 3.86 (6H, s); 3.93 (3H, s); 6.72 (1H, d, 9.4); 6.75 (1H, d, 2.2); 7.10 (2H, s); 8.27 (1H, dd, 9.4, 2.2). ^13^C-NMR δ: 37.9 (2) (CH_3_); 56.1 (2) (CH_3_); 60.7 (CH_3_); 105.0 (CH); 107.6 (2) (CH); 110.4 (CH); 131.2 (C); 142.5 (C); 146.1 (C); 148.1 (CH); 152.8 (2) (C); 159.3 (C); 194.9 (C). IR (film): 1659; 1595; 1411; 1328; 1125 cm^−1^. Mp (Hexane/Ethyl acetate): 101–102 °C. HRMS: calculated for C_17_H_21_N_2_O_4_ [M + H]^+^: 317.1501; found: 317.1498

#### 4.1.2. General Procedure to Obtain Diarylketones by Two-step Synthesis (**1c**, **1d**, **1e**, **1h**)

1.2 mmol per mmol of 1.6 M *n*BuLi in hexane was added to a stirred 0.2 M suspension of the corresponding bromo derivative in dry THF at −78 °C under inert atmosphere. After 1–2 h, the corresponding aromatic aldehyde was slowly added to the resulting yellowish solution, allowing the reaction to slowly reach room temperature. After 12–24 h, the mixture was poured over ice and extracted with CH_2_Cl_2_. The organic layers were dried over anhydrous Na_2_SO_4_, filtered, and evaporated under vacuum. Then, 1 mmol of PDC per mmol of crude and a small amount of tetrabutylammonium hydrogen sulfate were added in the dark to a 0.15–0.25 M solution of the alcohol in dry CH_2_Cl_2_ at 0 °C. The reaction was allowed to reach room temperature and proceed until completion (TLC) for 4–24 h. The reaction crude was filtered through silica, using CH_2_Cl_2_ and ethyl acetate as eluents, and the organic layers evaporated under vacuum to yield the diarylketones that were purified by column chromatography.

*(6-(Pyrrolidin-1-yl)pyridin-3-yl)(3,4,5-trimethoxyphenyl)methanone* (**1c**). 500 mg (2.84 mmol) of 6-(pyrrolidin-1-yl)nicotinaldehyde were added to a suspension of 840 mg (3.40 mmol) of 5-bromo-1,2,3-trimethoxybenzene and 2.6 mL (4.2 mmol) of *n*BuLi (1.6 M in hexane) in dry THF. After quenching and extraction of the crude, 393 mg (1.76 mmol) of PDC were added. After 4 h the crude was chromatographed CH_2_Cl_2_/EtOAc 1:1 to obtain 450 mg (1.31 mmol, 75%) of **1c**. ^1^H-NMR δ: 2.03 (4H, m); 3.54 (4H, m); 3.86 (6H, s); 3.89 (3H, s); 6.40 (1H, d, 8.8); 6.98 (2H, s); 7.98 (1H, d, 8.8); 8.62 (1H, s). ^13^C-NMR δ: 25.4 (2) (CH_2_); 47.0 (2) (CH_2_); 56.2 (2) (CH_3_); 60.9 (CH_3_); 106.0 (CH); 107.0 (2) (CH); 121.1 (C); 133.7 (C); 138.5 (CH); 141.0 (C); 152.8 (2) (C); 152.9 (CH); 158.3 (C); 193.1 (C). IR (KBr): 1640; 1548; 1330; 1127 cm^−1^. Mp (diethyl ether): 140–141 °C. HRMS: calculated for C_19_H_23_N_2_O_4_ [M + H]^+^: 343.1658; found: 343.1651.

*(4-(Dimethylamino)phenyl)(2,3,4-trimethoxyphenyl)methanone* (**1d**). 4.43 g (29.7 mmol) of 4-(dimethylamine)benzaldehyde were added to a suspension of 5.00 g (29.7 mmol) of 6-bromo-1,2,3-trimethoxybenzene and 18.6 mL (29.7 mmol) of *n*BuLi (1.6 M in hexane) in dry THF. After quenching and extraction of the crude, 16.8 g (44.7 mmol) of PDC were added. After 24 h the crude was chromatographed Hex/EtOAc 8:2 to obtain 1.86 g (5.90 mmol, 20%) of **1d**. ^1^H-NMR δ: 3.02 (6H, s); 3.76 (3H, s); 3.87 (6H, s); 6.60 (2H, d, 9.0); 6.67 (1H, d, 8.6); 7.01 (1H, d, 8.6); 7.72 (2H, d, 9.0). ^13^C-NMR δ: 40.1 (2) (CH_3_); 56.1 (CH_3_); 61.1 (CH_3_); 61.9 (CH_3_); 106.8 (CH); 110.5 (2) (CH); 124.2 (CH); 125.5 (C); 127.7 (C); 132.4 (2) (CH); 142.0 (C); 152.0 (C); 153.5 (C); 155.0 (C); 193.6 (C). IR (KBr): 1636; 1588 cm^−1^. Mp (Hexane/CH_2_Cl_2_): 205–207 °C. HRMS: calculated for C_18_H_21_NO_4_ [M + Na]^+^: 338.1368; found: 338.1360.

*(2,5-dimethoxyphenyl)(4-(dimethylamino)phenyl)methanone* (**1e**). 5.40 g (36.2 mmol) of 4-(dimethylamine)benzaldehyde were added to a suspension of 5.02 g (36.2 mmol) of 2-bromo-1,4-dimethoxybenzene and 22.6 mL (36.2 mmol) of *n*BuLi (1.6 M in hexane) in dry THF. After quenching and extraction of the crude, 22.05 g (58.6 mmol) of PDC were added. After 24 h the crude was chromatographed Hex/EtOAc 8:2 to obtain 3.52 g (12.40 mmol, 32%) of **1e**. ^1^H-NMR δ: 3.03 (6H, s); 3.68 (3H, s); 3.75 (3H, s); 6.61 (2H, d, 8.8); 6.85 (1H, d, 2.5); 6.90 (1H, dd, 8.8, 2.5); 6.92 (1H, d, 8.8); 7.74 (2H, d, 8.8). ^13^C-NMR δ: 40.0 (2) (CH_3_); 55.8 (CH_3_); 56.5 (CH_3_); 110.4 (2) (CH); 113.0 (CH); 114.1 (CH); 115.9 (CH); 125.1 (C); 130.9 (C); 132.3 (2) (CH); 150.9 (C); 153.3 (C); 153.5 (C); 194.0 (C). IR (KBr): 1637; 1588; 1544 cm^−1^. Mp (*t-*butylmethyl ether): 78–80 °C. HRMS: calculated for C_17_H_20_NO_3_ [M + H]^+^: 286.1443; found: 286.1420.

*(2-(Pyrrolidin-1-yl)pyridin-4-yl)(3,4,5-trimethoxyphenyl)methanone* (**1h**). 1.00 g (5.67 mmol) of 2-(pyrrolidin-1-yl)isonicotinaldehyde were added to a suspension of 2.15 g (8.51 mmol) of 5-bromo-1,2,3-trimethoxybenzene and 6.40 mL (10.2 mmol) of *n*BuLi (1.6 M in hexane) in dry THF. After quenching and extraction of the crude, 0.78 g (14.2 mmol) of PDC were added. After 4 h the crude was chromatographed with CH_2_Cl_2_/MeOH 98:2 to obtain 735 mg (2.15 mmol, 38%) of **1h**. ^1^H-NMR δ: 1.98 (4H, m); 3.43 (4H, m); 3.82 (6H, s); 3.89 (3H, s); 6.55 (1H, s); 6.60 (1H, d, 5.6); 7.07 (2H, s); 8.20 (1H, d, 5.6). ^13^C-NMR δ: 25.4 (2) (CH_2_); 46.8 (2) (CH_2_); 56.2 (2) (CH_3_); 60.9 (CH_3_); 106.0 (CH); 107.7 (2) (CH); 110.1 (CH); 131.2 (C); 146.1 (C); 148.1 (CH); 152.9 (2) (C); 155.0 (C); 157.1 (C); 195.1 (C). IR (film): 1650; 1598; 1538; 1456; 1328; 1127 cm^−1^. HRMS: calculated for C_19_H_23_N_2_O_4_ [M + H]^+^: 343.1658; found: 343.1642.

#### 4.1.3. General Procedure to Obtain Oxime Derivatives (**2a**–**h**)

3–15 mmol of NH_2_OH·HCl per mmol of diarylketone and 2–4 droplets of pyridine were added to a 0.02–0.06 M solution of the corresponding diarylketone in 10–25 mL of MeOH, and the reaction was refluxed for 12–48 h. After cooling, the solvent was evaporated off, the residue was re-dissolved in CH_2_Cl_2_, washed with brine, and the organic layers were dried, filtered, and evaporated.

*(4-(Dimethylamino)phenyl)(3,4,5-trimethoxyphenyl)methanone oxime* (**2a**) [16]. 560 mg (7.93 mmol) of hydroxylamine hydrochloride were added to 500 mg (1.59 mmol) of ketone **1a** in methanol to obtain, after crystallization in MeOH/EtOAc, 300 mg (0.91 mmol, 57%) of a 45:55 *Z/E* mixture of **2a**. While isomer *E* crystals are yellow, isomer *Z* crystals are green, so they were manually separated. **2a** (*E*): ^1^H-NMR (CD_3_OD) δ: 2.89 (6H, s); 3.65 (6H, s); 3.67 (3H, s); 6.61 (2H, s); 6.63 (2H, d, 8.9); 7.25 (2H, d, 8.9). ^13^C-NMR (CD_3_OD) δ: 40.5 (2) (CH_3_); 56.4 (2) (CH_3_); 61.1 (CH_3_); 107.0 (2) (CH); 111.7 (2) (CH); 121.6 (C); 132.4 (2) (CH); 135.0 (C); 139.8 (C); 152.1 (C); 154.1 (2) (C); 158.1 (C). Mp. (MeOH/EtOAc): 148–149 °C. HRMS: calculated for C_18_H_22_N_2_O_4_ [M + Na]^+^: 353.1472; found: 353.1477. **2a** (*E*): ^1^H-NMR (CD_3_OD) δ: 2.90 (6H, s); 3.68 (6H, s); 3.71 (3H, s); 6.49 (2H, s); 6.77 (2H, d, 8.9); 7.24 (2H, d, 8.9). ^13^C-NMR (CD_3_OD) δ: 40.5 (2) (CH_3_); 56.4 (2) (CH_3_); 61.1 (CH_3_); 107.8 (2) (CH); 112.3 (2) (CH); 121.6 (C); 129.7 (2) (CH); 135.0 (C); 139.8 (C); 152.1 (C); 154.1 (2) (C); 158.1 (C). IR (KBr): 3410; 1604; 1527; 1126 cm^−1^. Mp (MeOH/EtOAc): 163–164 °C. HRMS: calculated for C_18_H_22_N_2_O_4_ [M+Na]^+^: 353.1477; found: 353.1470.

*(6-(Pyrrolidin-1-yl)pyridin-3-yl)(3,4,5-trimethoxyphenyl)methanone oxime* (**2c**). 170 mg (2.46 mmol) of hydroxylamine hydrochloride were added to 56 mg (0.16 mmol) of ketone **1c** in methanol to obtain, after crystallization in MeOH, 15 mg (0.04 mmol, 25%) of **2c**. ^1^H-NMR δ: 2.13 (4H, m); 3.80 (4H, m); 3.88 (6H, s); 3.92 (3H, s); 6.68 (1H, d, 8.8); 6.99 (2H, s); 8.22 (1H, dd, 8.8, 2.0); 8.65 (1H, d, 2.0). IR (KBr): 3490; 1610; 1590; 1430; 1380; 1210 cm^−1^.

*(4-(Dimethylamino)phenyl)(2,3,4-trimethoxyphenyl)methanone oxime* (**2d**). 862 mg (12.4 mmol) of hydroxylamine hydrochloride were added to 346 mg (1.10 mmol) of ketone **1d** in methanol to obtain, after column chromatography Hex/EtOAc 7:3, 158 mg (0.48 mmol, 44%) of **2d**. ^1^H-NMR δ: 2.96 (6H, s); 3.77 (3H, s); 3.91 (6H, s); 6.65 (2H, d, 7.6); 6.77 (1H, d, 7.4); 6.87 (1H, d, 7.4); 7.37 (2H, d, 7.6). ^13^C-NMR δ: 40.3 (2) (CH_3_); 55.9 (CH_3_); 60.8 (CH_3_); 60.9 (CH_3_); 107.2 (CH); 108.3 (C); 112.1 (2) (CH); 120.2 (C); 123.7 (CH); 128.0 (2) (CH); 142.2 (C); 151.0 (C); 151.2 (C); 154.05 (C); 155.96(C). IR (KBr): 3490; 1523; 1601 cm^−1^. Mp (CH_2_Cl_2_/Hexane): 179–181 °C. HRMS: calculated for C_18_H_22_N_2_O_4_ [M + Na]^+^: 353.1477; found: 353.1469.

*(2,5-Dimethoxyphenyl)(4-(dimethylamino)phenyl)methanone oxime* (**2e**). 494 mg (7.1 mmol) of hydroxylamine hydrochloride were added to 223 mg (0.80 mmol) of ketone **1e** in methanol to obtain, after column chromatography Hex/EtOAc 8:2, 128 mg (0.40 mmol, 50%) of **2e**. ^1^H-NMR δ: 2.99 (6H, s, NCH_3_); 3.73 (3H, s, OCH_3_); 3.78 (3H, s, OCH_3_); 6.63 (2H, d, 8.0); 6.74 (1H, d, 7.6); 6.95 (1H, d, 7.6); 7.38 (2H, d, 9.0). ^13^C-NMR δ: 40.2 (2)(CH_3_); 55.7 (CH_3_); 56.7 (CH_3_); 111.7 (2)(CH); 113.0 (CH); 114.8 (CH); 115.0 (CH); 123.1 (C); 123.8 (C); 127.9 (2)(CH); 150.6 (C); 151.0 (C); 153.6 (C); 155.7 (C). IR (KBr): 3485, 1525, 1606 cm^−1^. Mp (CH_2_Cl_2_/hexane): 163–165 °C. HRMS: calculated for C_17_H_21_N_2_O_3_ [M + H]^+^: 301.1552; found: 301.1556.

*(3-(Dimethylamino)phenyl)(3,4,5-trimethoxyphenyl)methanone oxime* (**2f**). 220 mg (3.17 mmol) of hydroxylamine hydrochloride were added to 100 mg (0.32 mmol) of ketone **1f** in methanol to obtain 96 mg (0.29 mmol, 91%) of a 1:1 mixture of *Z*/*E* isomers of **2f**. (*Z*): ^1^H-NMR δ: 2.87 (6H, s); 3.72 (6H, s); 3.80 (3H, s); 6.55 (2H, s); 6.61 (1H, d, 8.0); 6.65 (1H, d, 8.0); 6.72 (1H, s); 7.26 (1H, t, 8.0). ^13^C-NMR δ: 40.6 (2) (CH_3_); 56.2 (2) (CH_3_); 60.9 (CH_3_); 106.7 (2) (CH); 113.1 (CH); 114.0 (CH); 117.3 (CH); 128.3 (C); 128.9 (CH); 133.3 (C); 136.9 (C); 150.3 (C); 152.9 (2) (C); 158.5 (C). (*E*): ^1^H-NMR δ: 2.90 (6H, s); 3.76 (6H, s); 3.84 (3H, s); 6.62 (1H, s); 6.71 (2H, s); 6.74 (1H, d, 8.0); 6.90 (1H, d, 8.0); 7.13 (1H, t, 8.0). ^13^C-NMR δ: 40.6 (2) (CH_3_); 56.2 (2) (CH_3_); 60.9 (CH_3_); 105.3 (2) (CH); 111.8 (CH); 113.4 (CH); 117.1 (CH); 128.3 (C); 128.9 (CH); 133.3 (C); 136.9 (C); 150.3 (C); 152.9 (2) (C); 158.1 (C). IR (film): 3428; 1582; 1503; 1411; 1344; 1236; 1127 cm^−1^. HRMS: calculated for C_18_H_23_N_2_O_4_ [M + H]^+^: 331.1658; found: 331.1650.

*(2-(Dimethylamino)pyridin-4-yl)(3,4,5-trimethoxyphenyl)methanone oxime* (**2g**). 40 mg (0.54 mmol) of hydroxylamine hydrochloride were added to 55 mg (0.17 mmol) of ketone **1g** in methanol to obtain, after column chromatography with Hex/EtOAc 1:1, 12 mg (0.04 mmol, 23%) of a 4:6 mixture of *Z*/*E* isomers of **2g**. (*Z*): ^1^H-NMR δ: 3.11 (6H, s); 3.86 (6H, s); 3.91 (3H, s); 6.61 (2H, s); 6.66 (1H, s); 8.13 (1H, d, 5.4); 8.25 (1H, d, 5.4). (***E***): ^1^H-NMR δ: 3.16 (6H, s); 3.79 (6H, s); 3.83 (3H, s); 6.47 (1H, s); 6.50 (1H, d, 5.4); 6.74 (2H, s); 8.22 (1H, d, 5.4). ^13^C-NMR δ: 38.6 (2) (CH_3_); 56.2 (2) (CH_3_); 60.9 (CH_3_); 104.1 (CH); 104.8 (2) (CH); 110.0 (CH); 126.7 (C); 130.4 (C); 146.1 (C); 142.3 (CH); 153.1 (2) (C); 156.3 (C); 158.9 (C). IR (film): 3205; 1650; 1586; 1458; 1236; 1125 cm^−1^. HRMS: calculated for C_17_H_22_N_3_O_4_ [M + H]^+^: 332.1610; found: 332.1609.

*(2-(Pyrrolidin-1-yl)pyridin-4-yl)(3,4,5-trimethoxyphenyl)methanone oxime* (**2h**). 167 mg (2.41 mmol) of hydroxylamine hydrochloride were added to 275 mg (0.80 mmol) of ketone **1h** in methanol to obtain, after column chromatography Hex/EtOAc 6:4, 120 mg (0.34 mmol, 42%) of a 4:6 mixture of *Z/E* isomers of **2h**. (*Z*): ^1^H-NMR δ: 1.60 (4H, m); 3.43 (4H, m); 3.83 (6H, s); 3.91 (3H, s); 6.46 (1H, d, 4.6); 6.60 (1H, s); 6.61 (2H, s); 8.14 (1H, d, 4.6). **(*E*)**: ^1^H-NMR δ: 2.02 (4H, m); 3.47 (4H, m); 3.80 (6H, s); 3.87 (3H, s); 6.30 (1H, s); 6.42 (1H, d, 4.6); 6.76 (2H, s); 8.26 (1H, d, 4.6). ^13^C-NMR δ: 25.5 (2) (CH_2_); 46.9 (2) (CH_2_); 56.2 (2) (CH_3_); 60.9 (CH_3_); 104.9 (2) (CH); 114.6 (CH); 119.5 (CH); 130.2 (C); 139.6 (C); 142.7 (C); 147.7 (CH); 153.0 (2) (C); 155.4 (C); 155.4 (C). IR (KBr): 3433; 1605; 1542; 1238; 1127 cm^−1^. HRMS: calculated for C_19_H_24_N_3_O_4_ [M + H]^+^: 358.1767; found: 358.1769.

#### 4.1.4. General Procedure to Obtain Isocombretastatines by Wittig Reactions (**3a**–**h**)

The required methyltriphenylphosphonium iodide (3 mmol/mmol of diarylketone) was suspended in dry THF (30–60 mL) and cooled to −40 °C under inert atmosphere, *n*Butyllithium (1.6 M in hexane, 0.9 equivalents respect to the salt) was added drop wise, and the resulting solution was stirred at this temperature for 60 min turning from deep-red to yellow-red. Then, a solution of the diarylketone in dry THF (15 mL) was added and warmed to room temperature. Once completed, the reaction mixture was poured onto ammonium chloride and extracted with ethyl acetate. The organic layers were washed with brine, dried over sodium sulfate and concentrated under vacuum. The residue was purified by column chromatography on silica gel.

*N,N-Dimethyl-4-(1-(3,4,5-trimethoxyphenyl)vinyl)aniline* (**3a**) [16]. A solution of 1.40 g (4.44 mmol) of **1a** in THF was added to a suspension of 5.40 g (13.3 mmol) of methyltriphenylphosphonium iodide and 6.90 mL (11.0 mmol) of *n*BuLi (1.6 M in hexane) in dry THF at −40 °C to obtain, after column chromatography with Hex/EtOAc 6:4, 1.05 g (3.35 mmol, 75%) of compound **3a**. ^1^H-NMR δ: 2.93 (6H, s); 3.80 (6H, s); 3.90 (3H, s); 5.24 (1H, d, 1.4); 5.36 (1H, d, 1.4); 6.62 (2H, s); 6.65 (2H, d, 8.6); 7.23 (2H, d, 8.6). ^13^C-NMR δ: 40.4 (2) (CH_3_); 56.1 (2) (CH_3_); 60.9 (CH_3_); 105.8 (2) (CH); 110.9 (CH_2_); 111.9 (2) (CH); 129.1 (2) (CH); 137.6 (C); 138.1 (2) (C); 149.9 (C); 150.3(C); 152.9 (2) (C). IR (film): 1605; 1579; 1516; 1350; 1126; 1008 cm^−1^. HRMS: calculated for C_19_H_24_NO_3_ [M + H]^+^: 314.1756; found: 314.1755.

*N,N-Dimethyl-5-(1-(3,4,5-trimethoxyphenyl)vinyl)pyridin-2-amine* (**3b**). A solution of 127 mg (0.40 mmol) of **1b** in THF was added to a suspension of 485 mg (1.20 mmol) of methyltriphenylphosphonium iodide and 0.23 mL (1.0 mmol) of *n*BuLi (1.6 M in hexane) in dry THF at −40 °C to obtain, after column chromatography with Hex/EtOAc 7:3, 51 mg (0.2 mmol, 41%) of compound **3b**. ^1^H-NMR δ: 3.12 (6H, s); 3.82 (6H, s); 3.86 (3H, s); 5.25 (1H, s); 5.33 (1H, s); 6.47 (1H, d, 8.8); 6.55 (2H, s); 7.40 (1H, dd, 8.8, 2.4); 8.20 (1H, d, 2.4). ^13^C-NMR δ: 38.3 (2) (CH_3_); 56.2 (2) (CH_3_); 61.0 (CH_3_); 105.2 (CH); 105.4 (2) (CH); 111.6 (C); 111.8 (CH_2_); 124.7 (C); 137.2 (C); 137.6 (CH); 146.5 (C); 147.0 (CH); 153.0 (2) (C); 158.3 (C). IR (KBr): 1603; 1577; 1510; 1454; 1388; 1125 cm^−1^. Mp (Hex/EtOAc)**:** 92–93 °C. HRMS: calculated for C_18_H_23_N_2_O_3_ [M + H]^+^: 315.1709; found: 315.1715.

*2-(Pyrrolidin-1-yl)-5-(1-(3,4,5-trimethoxyphenyl)vinyl)pyridine* (**3c**). A solution of 176 mg (0.51 mmol) of **1c** in THF was added to a suspension of 623 mg (1.54 mmol) of methyltriphenylphosphonium iodide and 1.0 mL (1.6 mmol) of *n*BuLi (1.6 M in hexane) in dry THF at −40 °C to obtain, after column chromatography with Hex/EtOAc 1:1, 80 mg (0.23 mmol, 46%) of compound **3c**. ^1^H-NMR δ: 2.00 (4H, m); 3.47 (4H, m); 3.79 (6H, s); 3.85 (3H, s); 5.23 (1H, s); 5.31 (1H, s); 6.33 (1H, d, 8.8); 6.54 (2H, s); 7.41 (1H, dd, 8.8, 2.2); 8.18 (1H, d, 2.2). ^13^C-NMR δ: 25.5 (2) (CH_2_); 46.9 (2) (CH_2_); 56.1 (2) (OCH_3_); 60.8 (OCH_3_); 105.5 (2) (CH); 106.0 (CH); 111.2 (CH_2_); 124.3 (C); 137.1 (CH); 134.5 (C); 137.8 (C); 147.0 (CH); 147.2 (C); 152.8 (2) (C); 156.3 (C). IR (KBr): 1600; 1506; 1235; 1126; 1006 cm^−1^. HRMS: calculated for C_20_H_25_N_2_O_3_ [M + H]^+^: 341.1865; found 341.1870.

*N,N-dimethyl-4-(1-(2,3,4-trimethoxyphenyl)vinyl)aniline* (**3d**). A solution of 500 mg (1.58 mmol) of **1d** in THF was added to a suspension of 1.93 g (4.80 mmol) of methyltriphenylphosphonium iodide and 2.5 mL (4.0 mmol) of *n*BuLi (1.6 M in hexane) in dry THF at −40 °C to obtain, after column chromatography with Hex/EtOAc 9:1, 349 mg (1.11 mmol, 70%) of compound **3d**. ^1^H-NMR δ: 2.95 (6H, s); 3.59 (3H, s); 3.87 (3H, s); 3.89 (3H, s); 5.09 (1H, d, 1.6); 5.54 (1H, d, 1.6); 6.64 (2H, d, 8.8); 6.67 (1H, d, 8.4); 6.92 (1H, d, 8.4); 7.19 (2H, d, 8.8). ^13^C-NMR δ: 40.5 (2) (CH_3_); 56.0 (CH_3_); 60.8 (CH_3_); 60.9 (CH_3_); 106.9 (CH); 111.8 (CH_2_); 112.1 (2) (CH); 125.4 (CH); 127.6 (2) (CH); 129.8 (C); 129.9 (C); 142.4 (C); 146.5 (C); 150.1 (C); 151.8 (C); 153.3 (C). IR (KBr): 1602; 1521 cm^−1^. Mp (*t*-butylmethylether)**:** 63–65 °C. HRMS: calculated for C_19_H_24_NO_3_ [M + H]^+^: 314.1756; found: 314.1748.

*4-(1-(2,5-Dimethoxyphenyl)vinyl)-N,N-dimethylaniline* (**3e**). A solution of 500 mg (1.75 mmol) of **1e** in THF was added to a suspension of 2.12 g (4.80 mmol) of methyltriphenylphosphonium iodide and 2.7 mL (4.38 mmol) of *n*BuLi (1.6 M in hexane) in dry THF at −40 °C to obtain, after column chromatography with Hex/EtOAc 6:4, 245 mg (0.87mmol, 50%) of compound **3e**. ^1^H-NMR δ**:** 2.99 (6H, s); 3.68 (3H, s); 3.82 (3H, s); 5.18 (1H, s); 5.70 (1H, s); 6.70 (2H, d, 9.0); 6.90 (3H, s); 7.28 (2H, d, 9.0). ^13^C-NMR δ: 40.6 (2)(CH_3_); 55.8 (CH_3_); 56.7 (CH_3_); 111.7 (CH_2_); 112.1 (2) (CH); 112.8 (CH); 113.2 (CH); 117.1 (CH); 127.3 (2) (CH); 128.8 (C); 133.0 (C); 146.4 (C); 150.1 (C); 151.6 (C); 153.7 (C). IR (KBr): 1525, 1605 cm^−1^. Mp (*t*-butylmethylether)**:** 68–70 °C. HRMS: calculated for C_18_H_22_NO_2_ [M + H]^+^: 284.1651; found: 284.1645.

*N,N-Dimethyl-3-(1-(3,4,5-trimethoxyphenyl)vinyl)aniline* (**3f**). A solution of 300 mg (0.95 mmol) of **1f** in THF was added to a suspension of 1.15 g (2.85 mmol) of methyltriphenylphosphonium iodide and 1.2 mL (1.9 mmol) of *n*BuLi (1.6 M in hexane) in dry THF at −40 °C to obtain, after column chromatography with Hex/EtOAc 7:3, 280 mg (0.89mmol, 94%) of compound **3f**. ^1^H-NMR (CD_3_OD) δ**:** 2.90 (6H, s); 3.75 (6H, s); 3.78 (3H, s); 5.38 (1H, d, 1.2); 5.41 (1H, d, 1.2); 6.61 (2H, s); 6.65 (1H, d, 8.0); 6.73 (1H, s); 6.75 (1H, dd, 8.0); 7.16 (1H, t, 8.0). ^13^C-NMR (CD_3_OD) δ**:** 40.8 (2) (CH_3_); 56.2 (2) (CH_3_); 60.9 (CH_3_); 105.7 (2) (CH); 112.3 (CH); 112.8 (CH); 113.6 (CH_2_); 117.3 (CH); 128.8 (CH); 137.5 (C); 137.7 (C); 142.1 (C); 150.5 (C); 150.8 (C); 152.8 (2) (C). IR (film): 1596; 1578; 1503; 1411; 1353; 1235; 1127; 898 cm^−1^. HRMS: calculated for C_19_H_24_NO_3_ [M + H]^+^: 314.1756; found: 314.1750

*N,N-Dimethyl-4-(1-(3,4,5-trimethoxyphenyl)vinyl)pyridin-2-amine* (**3g**). A solution of 141 mg (0.44 mmol) of **1g** in THF was added to a suspension of 510 mg (1.26 mmol) of methyltriphenylphosphonium iodide and 1.0 mL (1.6 mmol) of *n*BuLi (1.6 M in hexane) in dry THF at −40 °C to obtain, after column chromatography with CH_2_Cl_2_/EtOAc 9:1, 102 mg (0.32 mmol, 73%) of compound **3g**. ^1^H-NMR (CD_3_OD) δ: 3.10 (6H, s); 3.82 (6H, s); 3.88 (3H, s); 5.51 (1H, s); 5.53 (1H, s); 6.49 (1H, d, 8.2); 6.52 (1H, s); 6.53 (2H, s); 8.12 (1H, d, 8.2). ^13^C-NMR (CD_3_OD) δ**:** 37.1 (2) (CH_3_); 55.0 (2) (CH_3_); 59.5 (CH_3_); 105.1 (2) (CH); 110.9 (CH); 111.9 (CH); 114.9 (CH_2_); 135.8 (C); 137.6 (C); 146.5 (CH); 148.6 (C); 150.3 (C); 152.6 (2) (C); 159.3 (C). IR (film): 1594; 1541; 1502; 1409; 1234; 1127 cm^−1^. HRMS: calculated for C_18_H_23_N_2_O_3_ [M + H]^+^: 315.1709; found: 315.1712.

*2-(Pyrrolidin-1-yl)-4-(1-(3,4,5-trimethoxyphenyl)vinyl)pyridine* (**3h**). A solution of 360 mg (1.05 mmol) of **1g** in THF was added to a suspension of 1.27 g (3.15 mmol) of methyltriphenylphosphonium iodide and 1.6 mL (2.6 mmol) of *n*BuLi (1.6 M in hexane) in dry THF at −40 °C to obtain, after column chromatography with Hex/EtOAc 8:2, 295 mg (0.87 mmol, 83%) of compound **3h**. ^1^H-NMR δ: 1.98 (4H, m); 3.43 (4H, m); 3.80 (6H, s); 3.86 (3H, s); 5.47 (1H, s); 5.50 (1H, s); 6.31 (2H, s); 6.46 (1H, d, 5.0); 6.53 (1H, s); 8.10 (1H, d, 5.0). ^13^C-NMR δ**:** 25.5 (2) (CH_2_); 46.8 (2) (CH_2_); 56.1 (2) (CH_3_); 60.9 (CH_3_); 105.5 (2) (CH); 105.8 (CH); 111.1 (CH); 115.3 (CH_2_); 135.9 (C); 137.9 (C); 147.9 (C); 149 (CH); 149.7 (C); 152.9 (2) (C); 157.6 (C). IR (film): 1593; 1535; 1492; 1454; 1236; 1126 cm^−1^. HRMS: calculated for C_20_H_25_N_2_O_3_ [M + H]^+^: 341.1865; found: 341.1862.

#### 4.1.5. General Procedure to Obtain Dihydro Derivatives by Reduction of Isocombretastatins (**4d**, **4e**, **4f**)

Catalytic amounts of Pd/C were added over a 0.01–0.02 M solution of the isocombretastatins in ethanol. After 24 h stirring at room temperature and under hydrogen atmosphere, the residue was filtered over silica gel and concentrated under vacuum.

*N,N-Dimethyl-4-(1-(2,3,4-trimethoxyphenyl)ethyl)aniline* (**4d**). Ten mg of Pd/C were added to a solution of 84 mg (0.30 mmol) of **3d** in EtOH under hydrogen atmosphere to obtain, after column chromatography with Hex/EtOAc 95:5, 56 mg (0.20 mmol; 67%) of **4d**. ^1^H-NMR δ: 1.55 (3H, d, 7.3); 2.91 (6H, s); 3.70 (3H, s); 3.84 (3H, s); 3.87 (3H, s); 4.40 (1H, q, 7.3); 6.63 (1H, d, 8.6); 6.71 (2H, d, 8.6); 6.88 (1H, d, 8.6); 7.12 (2H, d,8.6). ^13^C-NMR δ**:** 21.8 (CH); 36.7 (CH_3_); 40.8 (CH); 55.9 (CH); 60.6 (CH); 60.8 (CH); 107.0 (CH_3_); 112.8 (CH_3_); 121.7 (CH_3_); 128.1 (2)(CH_3_); 133.3 (C); 135.2 (C); 142.2 (C); 148.7 (C); 151.4 (C); 151.7 (C). IR (film): 1519; 1610 cm^−1^. HRMS: calculated for C_19_H_26_NO_3_ [M + H]^+^: 316.1913; found: 316.1915.

*4-(1-(2,5-Dimethoxyphenyl)ethyl)-N,N-dimethylaniline* (**4e**). Ten mg of Pd/C were added to a solution of 63.5 mg (0.22 mmol) of **3e** in EtOH under hydrogen atmosphere to obtain, after column chromatography with Hex/EtOAc 95:5, 44 mg (0.15 mmol; 68%) of **4e**. ^1^H-NMR δ: 1.55 (3H, d, 7.2); 2.91 (6H, s); 3.73 (3H, s); 3.75 (3H, s); 4.49 (1H, q, *7.*2); 6.56–6.79 (5H, m); 7.14 (2H, d, 8.6). ^13^C-NMR δ**:** 20.9 (CH_3_); 36.5 (CH); 40.9 (CH_3_); 55.5 (CH_3_); 56.2 (CH_3_); 77.0 (c); 110.2 (CH); 111.6 (CH); 112.9 (CH); 114.6 (CH); 128.2 (2) (CH); 137.2 (C); 148.7 (C); 151.2 (C); 153.6 (C). IR (film): 1517; 1613 cm^−1^. HRMS: calculated for C_18_H_24_NO_2_ [M + H]^+^: 286.1807; found: 286.1816.

*N,N-Dimethyl-3-(1-(3,4,5-trimethoxyphenyl)ethyl)aniline* (**4f**). Ten mg of Pd/C were added to a solution of 50 mg (0.16 mmol) of **3f** in EtOH under hydrogen atmosphere to obtain 47 mg (0.15 mmol; 94%) of **4f**. ^1^H-NMR δ: 1.63 (3H, d, 7.5); 2.93 (6H, s); 3.99 (9H, s); 4.10 (1H, q, 7.5); 6.48 (2H, s); 6.60 (1H, d, 6.5); 6.62 (1H, d, 6.5); 6.66 (1H, bs); 7.22 (1H, t, 6.5). ^13^C-NMR δ: 22.2 (CH_3_); 41.1 (2) (CH_3_); 45.5 (CH); 56.1 (2) (CH_3_); 60.9 (CH_3_); 104.7 (2) (CH); 111.1 (CH); 112.7 (CH); 129.2 (2) (CH); 136.2 (C); 142.2 (C); 147.2 (C); 150.2 (C); 153.0 (2) (C). IR (film): 1589; 1499; 1458; 1327; 1233; 1126 cm^−1^. HRMS: calculated for C_19_H_26_NO_3_ [M + H]^+^: 316.1913; found: 316.1909.

#### 4.1.6. General Procedure to Obtain Combretastatins by Wittig Reactions (**8a**–**c** and **8g**–**i**)

0.75 to 1.10 mmol per mmol of *n*BuLi (1.6 M in hexane) were added to a 0.08 M suspension of triphenyl(3,4,5-trimethoxybenzyl)phosphonium bromide in dry THF at −78 °C under inert atmosphere. After one hour stirring, 0.25 to 0.90 mmol of the corresponding aromatic aldehyde per mmol of the phosphonium salt were added and reaction was allowed to reach room temperature. After 24 h, the reaction was poured onto ice and extracted with CH_2_Cl_2_. Organic layers were washed with brine, dried over sodium sulphate and concentrated under vacuum. The residue was purified by column chromatography on silica gel.

*N,N-dimethyl-4-(3,4,5-trimethoxystyryl)aniline* (**8a**) [15]. A solution of 1.00 g (6.70 mmol) of *p*-dimethylaminebenzaldehyde in THF was added to a suspension of 4.20 g (8.04 mmol) of triphenyl(3,4,5-trimethoxybenzyl)phosphonium bromide and 6.10 mL (9.65 mmol) of *n*BuLi (1.6 M in hexane) in dry THF at −78 °C to obtain, after column chromatography with Hex/EtOAc 7:3, 290 mg (0.92 mmol, 14%) of isomer (*Z*)-**8a**, 113 mg (0.36 mmol, 5%) of isomer (*E*)-**8b** and 1.20 g (3.83 mmol, 57%) of a 1:1 *Z*/*E* mixture. (*Z*)-**8a:** ^1^H-NMR δ: 2.93 (6H, s); 3.71 (6H, s); 3.85 (3H, s); 6.32 (1H, d, 12.2); 6.46 (1H, d, 12.2); 6.57 (2H, s); 6.60 (2H, d; 8.6); 7.20 (2H, d, 8.6). ^13^C-NMR δ**:** 40.5 (2) (CH_3_); 55.9 (2) (CH_3_); 60.9 (CH_3_); 105.8 (2) (CH); 112.0 (2) (CH); 125.1 (C); 126.8 (CH); 130.1 (3) (CH); 133.6 (C); 136.7 (C); 149.7 (C); 152.9 (2) (C). IR (film): 1606; 1585; 1516; 1236; 1126; 767 cm^−1^. HRMS: calculated for C_19_H_24_NO_3_ [M + H]^+^: 314.1751; found: 314.1758. (*E*)-**8a:** ^1^H-NMR δ: 2.99 (6H, s); 3.87 (3H, s); 3.92 (6H, s); 6.74 (2H, s); 6.76 (2H, d, 8.8); 6.82 (1H, d, 15.4); 6.96 (1H, d, 15.4); 7.40 (2H, d, 8.8).^13^C-NMR δ**:** 40.6 (2) (CH_3_); 56.1 (2) (CH_3_); 61.0 (CH_3_); 103.0 (2) (CH); 112.6 (2) (CH); 124.4 (CH); 125.8 (C); 127.6 (2) (CH); 128.4 (CH); 134.0 (C); 137.2 (C); 150.0 (C); 153.4 (2) (C). IR (film): 1606; 1585; 1516; 1236; 1126; 767 cm^−1^. Mp (diethyl ether): 94–95 °C. HRMS: calculated for C_19_H_24_NO_3_ [M + H]^+^: 314.1756; found: 314.1760.

*N,N-dimethyl-5-(3,4,5-trimethoxystyryl)pyridin-2-amine* (**8b**). A solution of 230 mg (1.53 mmol) of 6-(dimethylamino)nicotinaldehyde in THF was added to a suspension of 1.30 g (2.14 mmol) of triphenyl(3,4,5-trimethoxybenzyl)phosphonium bromide and 1.5 mL (2.10 mmol) of *n*BuLi (1.6 M in hexane) in dry THF at −40 °C to obtain, after column chromatography with Hex/EtOAc 1:1, 65 mg (0.21 mmol, 14%) of isomer (*Z*)-**8b**, and 213 mg (0.68 mmol, 44%) of a 1:1 *Z/E* mixture. (*Z*)-**8b:** ^1^H-NMR δ: 3.05 (6H, s); 3.72 (6H, s); 3.83 (3H, s); 6.32 (1H, d, 8.8); 6.38 (2H, s); 6.51 (2H, s); 7.38 (1H, dd, 8.8, 2.2); 8.09 (1H, d, 2.2). ^13^C-NMR δ**:** 38.1 (2) (CH_3_). 56.0 (2) (CH_3_). 60.8 (CH_3_). 104.7 (CH). 105.7 (2) (CH). 109.5 (C). 120.4 (C). 126.9 (CH). 127.8 (CH). 133.2 (C). 133.6 (C). 137.2 (CH). 148.9 (CH). 153.3 (2) (C). 158.5 (C). IR (film): 1606; 1600; 1508; 1457; 1389; 1236; 1126 cm^−1^. HRMS: calculated for C_18_H_23_N_2_O_3_ [M + H]^+^: 315.1709; found: 315.1708. (***E***)-**8b**: ^1^H-NMR δ: 3.11 (6H, s); 3.86 (3H, s); 3.92 (6H, s); 6.52 (1H, d, 8.8); 6.69 (2H, s); 6.80 (1H, d, 16.4); 6.89 (1H, d, 16.4); 7.67 (1H, dd, 8.8, 2.2); 8.22 (1H, d, 2.2). ^13^C-NMR δ**:** 38.5 (2) (CH_3_); 56.4 (2) (CH_3_); 61.2 (CH_3_); 103.2 (2) (CH); 106.2 (CH); 121.3 (C); 125.5 (CH); 128.1 (CH); 133.8 (C); 134.1 (CH); 137.6 (CH); 147.5 (C); 153.6 (2) (C); 158.8 (C). IR (film): 1606; 1585; 1516; 1236; 1126; 767 cm^−1^.

*2-(Pyrrolidin-1-yl)-5-(3,4,5-trimethoxystyryl)pyridine* (**8c**). A solution of 57 mg (0.32 mmol) of 6-(pyrrolidin-1-yl)nicotinaldehyde in THF was added to a suspension of 156 mg (0.27 mmol) of triphenyl(3,4,5-trimethoxybenzyl)phosphonium bromide and 0.2 mL (0.12 mmol) of *n*BuLi (1.6 M in hexane) in dry THF at −40 °C to obtain, after column chromatography with Hex/EtOAc 1:1, 40 mg (0.12 mmol, 38%) of a 1:1 *Z*/*E* mixture of compound **8c**. (*Z*)-**8c**: ^1^H-NMR δ: 2.04 (4H, m); 3.50 (4H, m); 3.78 (6H, s); 3.92 (3H, s); 6.23 (1H, d, 8.8); 6.33 (1H, d, 12.0); 6.43 (1H, d, 12.0); 6.49 (2H, s); 7.40 (1H, dd, 8.8, 2.2); 8.11 (1H, d, 2.2). (*E*)-**8c:** 2.02 (4H, m); 3.50 (4H, m); 3.85 (6H, s); 3.93 (3H, s); 6.36 (1H, s); 6.70 (2H, s); 6.71 (1H, d, 4.4); 6.84 (1H, d, 2.2); 6.94 (1H, s); 8.22 (1H, d, 2.2). HRMS: calculated for C_20_H_25_N_2_O_3_ [M + H]^+^: 341.1865; found: 341.1868.

*2-(Pyrrolidin-1-yl)-4-(3,4,5-trimethoxystyryl)pyridine* (**8h**). A solution of 400 mg (2.27 mmol) of 6-(dimethylamino)nicotinaldehyde in THF was added to a suspension of 1.66 g (3.18 mmol) of triphenyl(3,4,5-trimethoxybenzyl)phosphonium bromide and 2.0 mL (3.18 mmol) of *n*BuLi (1.6 M in hexane) in dry THF at −40 °C to obtain, after column chromatography with Hex/EtOAc 6:4, 123 mg (0.23 mmol, 16%) of isomer (*Z*)-**8h**, and 181 mg (0.53 mmol, 17%) of a 1:1 *Z/E* mixture. (*Z*)-**8h:** ^1^H-NMR δ: 1.95 (4H, m); 3.35 (4H, m); 3.68 (6H, s); 3.82 (3H, s); 6.24 (1H, d, 1.4); 6.40 (1H, d, 12.2); 6.41 (1H, dd, 5.2, 1.4); 6.50 (2H, s); 6.54 (1H, d, 12.2); 8.02 (1H, d, 5.2).^13^C-NMR δ**:** 25.5 (2) (CH_2_); 46.6 (2) (CH_2_); 56.0 (2) (CH_3_); 60.9 (CH_3_); 106.1 (2) (CH); 106.1 (CH); 111.3 (CH); 128.5 (CH); 132.2 (C); 132.3 (CH); 137.5 (C) 145.9 (C); 147.9 (2) (C); 152.9 (CH); 157.6 (C). IR (film): 1650; 1598; 1538; 1329; 1229; 1127 cm^−1^. HRMS: calculated for C_20_H_25_N_2_O_3_ [M + H]^+^: 341.1865; found: 341.1868. (*E*)-**8h:** ^1^H-NMR δ: 1.98 (4H, m); 3.45 (4H, m); 3.80 (6H, s); 3.84 (3H, s); 6.48 (1H, s); 6.66 (1H, d, 5.4); 6.88 (1H, d, 16.1); 6.72 (2H, s); 7.08 (1H, d, 16.1); 8.07 (1H, d, 5.4). ^13^C-NMR δ**:** 25.6 (2) (CH_2_); 46.8 (2) (CH_2_); 56.1 (2) (CH_3_); 60.9 (CH_3_); 104.0 (2) (CH); 104.5 (CH); 106.2 (CH); 126.6 (C); 126.7 (CH); 131.8 (C); 132.0 (CH); 137.5 (CH) 145.6 (C); 152.8 (2) (C); 155.2 (C). IR (film): 1650; 1598; 1538; 1329; 1229; 1127 cm^−1^.

*1-(4-(3,4,5-Trimethoxystyryl)phenyl)pyrrolidine* (**8i**). A solution of 2.00 g (11.4 mmol) of 4-(pyrrolidin-1-yl)benzaldehyde in THF was added to a suspension of 7.10 g (13.70 mmol) of triphenyl(3,4,5-trimethoxybenzyl)phosphonium bromide and 9.8 mL (13.70 mmol) of *n*BuLi (1.6 M in hexane) in dry THF at −40 °C to obtain, after column chromatography with Hex/EtOAc 7:3, 1.83 g (5.40 mmol, 47%) of isomer (*Z*)-**8i**, and 1.20 g (0.53 mmol, 31%) of isomer (*E*)-**8i**. (*Z*)-**8i:** ^1^H-NMR δ: 1.99 (4H, m); 3.26 (4H, m, NCH_2_); 3.73 (6H, s, OCH_3_); 3.86 (3H, s, OCH_3_); 6.30 (1H, d; 12); 6,41 (2H, d; 8.2); 6.45 (1H, d, 12); 6.62 (2H, s); 7.21 (2H, d, 8.2). ^13^C-NMR δ: 25.5 (2) (CH_2_); 47.6 (2) (CH_2_); 56.0 (2) (OCH_3_); 60.9 (OCH_3_); 105.8 (2) (CH); 111.2 (2) (CH); 124.0 (C); 126.0 (CH); 130.3 (2) (CH); 130.4 (CH); 133.9 (C); 136.7 (C); 147.1 (C); 153.0 (2) (C). IR (film): 1604; 1237; 1126; 772 cm^−1^. HRMS: calculated for C_21_H_26_NO_3_ [M + H]^+^: 340.1913; found: 341.1902. (*E*)-**8i**: ^1^H-NMR δ: 2.00 (4H, m); 3.31 (4H, m, NCH_2_); 3.87 (3H, s, OCH_3_); 3.91 (6H, s, OCH_3_); 6.56 (2H, d, 8.6); 6.70 (2H, s); 6.80 (1H, d, 16.1); 6.98 (1H, d, 16.1); 7.40 (2H, d, 8.6). ^13^C-NMR δ**:** 25.5 (2) (CH_2_); 47.7 (2) (CH_2_); 56.1 (2) (OCH_3_); 61.0 (OCH_3_); 102.9 (2) (CH); 111.9 (2) (CH); 123.6 (CH); 124.7 (C); 127.7 (2) (CH); 128.7 (CH); 134.2 (C); 137.1 (C); 147.5 (C); 153.4 (2) (C). IR (film): 1585, 1233, 1126, 818 cm^−1^. Mp (EtOAc): 118–119 °C.

#### 4.1.7. General Procedure to Obtain the Ammonium Salt Derivatives (**5a**, **5d**, **5e**, **6a**, **7a**, **7d**, **9a**, **9b** and **9i**)

3.0–10.0 mmol per mmol of iodomethane were added to a 0.05 M solution of the corresponding amino derivatives in dry acetone and the reaction was refluxed in a sealed tube for 24 h. After cooling, the precipitate was filtered off and washed with ether.

*N,N,N-Trimethyl-4-(3,4,5-trimethoxybenzoyl)benzenaminiumiodide* (**5a**). 200 mg (0.63 mmol) of diarylketone **1a** and 0.237 mL (3.81 mmol) of iodomethane led to 130 mg (0.39 mmol, 62%) of **5a**. ^1^H-NMR (CD_3_OD) δ: 3.68 (9H, s); 3.75 (6H, s); 3.77 (3H, s); 6.99 (2H, s); 7.88 (2H, d, 6.8); 8.01 (2H, d, 6.8). ^13^C-NMR (CD_3_OD) δ**:** 56.9 (3) (CH_3_); 57.3 (2) (CH_3_); 64.5 (CH_3_); 109.1 (2) (CH); 121.5 (2) (CH); 132.7 (2) (CH); 141.0 (C); 147.8 (C); 150.8 (C); 153.8 (C); 154.5 (2) (C); 190.0 (C). IR (KBr): 3002; 1661; 1582; 1332; 1125 cm^−1^. HRMS: calculated for C_19_H_24_NO_4_ [M]^+^: 330.1700; found: 330.1690.

*N,N,N-Trimethyl-4-(2,3,4-trimethoxybenzoyl)benzenaminium iodide* (**5d**). 250 mg (0.79 mmol) of diarylketone **1d** and 0.495 mL (7.90 mmol) of iodomethane led to 180 mg (0.39 mmol, 69%) of **5d**, after crystallization in methanol. ^1^H-NMR (Pyridine) δ: 3.35 (3H, s); 3.40 (3H, s); 3.45 (3H, s); 3.74 (9H, s); 6.47 (1H, d, 8.8); 7.02 (1H, d, 8.8); 7.70 (2H, d, 8.4); 7.72 (2H, d, 8.4). ^13^C-NMR (Pyridine) δ**:** 54.8 (CH_3_); 56.8 (3) (CH_3_); 59.4 (CH_3_); 60.6 (CH_3_); 106.6 (CH); 119.6 (2) (CH); 124.1 (CH); 124.6 (C); 130.2 (2) (CH); 139.4 (C); 141.0 (C); 148.7 (C); 151.9 (C); 156.2 (C); 192.2 (C). IR (KBr): 1648; 1592; 1461; 1412; 1296; 1094 cm^−1^. Mp (methanol): 172–173 °C. HRMS: calculated for C_19_H_24_NO_4_ [M]^+^: 330.1700; found: 330.1705.

*4-(2,5-Dimethoxybenzoyl)-N,N,N-trimethylbenzenaminium iodide* (**5e**). 260 mg (0.91 mmol) of diarylketone **1e** and 0.170 mL (2.71 mmol) of iodomethane led to 135 mg (0.47 mmol, 52%) of **5e**, after crystallization in methanol. ^1^H-NMR (DMSO-*d*_6_) δ: 3.60 (3H, s); 3.63 (9H, s); 3.73 (3H, s); 6.93 (1H, t, 1.8); 7.15 (2H, d, 1.8); 7.84 (2H, d, 9.0); 8.08 (2H, d, 9.0). ^13^C-NMR (DMSO-*d*_6_) δ**:** 40.4 (3) (CH_3_); 56.3 (CH_3_); 56.4 (CH_3_); 113.6 (CH); 114.0 (CH); 117.8 (CH); 121.3 (2) (CH); 127.9 (C); 130.6 (2) (CH); 138.0 (C); 150.2 (C); 150.8 (C); 153.1 (C); 194.1 (C). IR (KBr): 1662; 1600; 1496; 1413; 1226; 1035 cm^−1^. Mp (diethyl ether): 162–163 °C. HRMS: calculated for C_18_H_22_NO_3_ [M]^+^: 300.1594; found: 330.1600.

*4-((Hydroxyimino)(3,4,5-trimethoxyphenyl)methyl)-N,N,N-trimethylbenzenaminium iodide* (**6a**). 100 mg (0.30 mmol) of oxime **2a** and 0.200 mL (3.03 mmol) of iodomethane led to 31 mg (0.09 mmol, 30%) of **6a**, after crystallization in methanol/ethyl acetate. (*Z*): ^1^H-NMR (CD_3_OD) δ: 3.28 (9H, s); 3.73 (6H, s); 3.78 (3H, s); 6.71 (2H, s); 7.62 (2H, d, 8.8); 8.01 (2H, d, 8.8). (*E*): ^1^H-NMR (CD_3_OD) δ: 3.30 (9H, s); 3.68 (6H, s); 3.78 (3H, s); 6.60 (2H, s); 7.71 (2H, d, 8.8); 7.88 (2H, d, 8.8). IR (KBr): 3470; 1582; 1505; 1344; 1130 cm^−1^. Mp (EtOAc/MeOH): 179–180 °C. HRMS: calculated for C_19_H_25_N_2_O_4_ [M]^+^: 345.1808; found: 345.1806.

*N,N,N-trimethyl-4-(1-(3,4,5-trimethoxyphenyl)vinyl)benzenaminium iodide* (**7a**). 250 mg (0.83 mmol) of isocombretastatine **3a** and 0.500 mL (7.97 mmol) of iodomethane led to 260 mg (0.79 mmol, 95%) of **7a.** ^1^H-NMR (CD_3_OD) δ: 3.62 (9H, s); 3.68 (6H, s); 3.73 (3H, s); 5.59 (1H, s); 5.63 (1H, s); 6.53 (2H, s); 7.53 (2H, d, 8.3); 7.93 (2H, d, 8.3). ^13^C-NMR (CD_3_OD) δ**:** 56.7 (3) (CH_3_); 57.8 (2) (CH_3_); 61.1 (CH_3_); 106.8 (2) (CH); 116.9 (CH_2_); 121.2 (2) (CH); 131.0 (2) (CH); 137.7 (C); 144.8 (C); 145.9 (C); 147.7 (C); 149.5 (C); 154.4 (2) (C). IR (KBr): 1662; 1600; 1496; 1413; 1226; 1035 cm^−1^. Mp (acetone): 176–177 °C. HRMS: calculated for C_20_H_26_NO_3_ [M]^+^: 328.1907; found: 328.1899.

*N,N,N-Trimethyl-4-(1-(2,3,4-trimethoxyphenyl)vinyl)benzenaminium iodide* (**7d**). 170 mg (0.54 mmol) of isocombretastatine **3d** and 0.170 mL (2.71 mmol) of iodomethane led to 160 mg (0.49 mmol, 91%) of **7d**, after crystallization in methanol. ^1^H-NMR (DMSO-*d*_6_) δ: 3.35 (3H, s); 3.51 (9H, s); 3.66 (3H, s); 3.76 (3H, s); 5.32 (1H, s); 5.70 (1H, s); 6.79 (1H, d, 8.2); 6.89 (1H, d, 8.2); 7.37 (2H, d, 8.6); 7.76 (2H, d, 8.6). ^13^C-NMR (DMSO-*d*_6_) δ**:** 55.9 (CH_3_); 56.4 (3) (CH_3_); 60.4 (2) (CH_3_); 107.9 (CH); 117.7 (CH_2_); 120.3 (2) (CH); 124.8 (CH); 127.2 (C); 127.4 (2) (CH); 141.8 (C); 142.6 (C); 144.6 (C); 146.1 (C); 150.9 (C); 153.6 (C). IR (KBr): 1594; 1496; 1459; 1406; 1295; 1090 cm^−1^. Mp (methanol): 168–169 °C. HRMS: calculated for C_20_H_26_NO_3_ [M]^+^: 328.1907; found: 328.1913.

*N,N,N-trimethyl-4-(3,4,5-trimethoxystyryl)benzenaminium iodide* (**9a**). 290 mg (0.92 mmol) of a 1:1 *Z*/*E* mixture of combretastatine **8a** and 0.500 mL (7.97 mmol) of iodomethane led to 260 mg (0.79 mmol, 86%) of a 7:3 *Z*/*E* mixture of compound **9a.** After crystallization in acetone/ethyl acetate, 61 mg (0.19 mmol, 21%) of isomer *Z* were isolated. ^1^H-NMR δ: 3.65 (6H, s); 3.79 (3H, s); 3.95 (9H, s); 6.37 (2H, s); 6.48 (1H, d, 12.2); 6.64 (1H, d, 12.2); 7.45 (2H, d, 8.6); 7.84 (2H, d, 8.6). ^13^C-NMR δ**:** 56.2 (2) (CH_3_); 57.9 (3) (CH_3_); 60.9 (CH_3_); 105.8 (2) (CH); 120.0 (2) (CH); 127.2 (CH); 131.0 (2) (CH). 132.9 (CH); 138.4 (C); 139.6 (C); 139.8 (C); 145.4 (C); 153.4 (2) (C). IR (KBr): 1581; 1240; 1125; 844 cm^−1^. Mp (acetone/ethylacetate): 75 °C. HRMS: calculated for C_20_H_26_NO_3_ [M]^+^: 328.1907; found: 328.1916.

*N,N,N-trimethyl-5-(3,4,5-trimethoxystyryl)pyridin-2-aminium iodide* (**9b**). 160 mg (0.51 mmol) of a 1:1 *Z*/*E* mixture of combretastatin **8b** and 0.320 mL (5.1 mmol) of iodomethane led to 213 mg of reaction crude. After crystallization in acetone/methanol, 35 mg (0.11 mmol, 22%) of isomer (*Z*)-**9b** were isolated. ^1^H-NMR (CD_3_OD) δ: 3.56 (9H, s); 3.75 (3H, s); 3.79 (6H, s); 6.84 (2H, s); 7.18 (1H, d, 1.0); 7.34 (1H, d, 16.0); 7.79 (1H, d, 8.6); 8.17 (1H, dd, 8.6, 2.2); 8.64 (1H, d, 2.2). IR (KBr): 1579; 1462; 1244; 1122 cm^−1^. HRMS: calculated for C_19_H_25_N_2_O_3_ [M]^+^: 329.1860; found: 329.1850.

*1-Methyl-1-(4-(3,4,5-trimethoxystyryl)phenyl)pyrrolidin-1-ium iodide* (**9i**). 100 mg (0.29 mmol) of combretastatin (*Z*)-**8i** and 0.182 mL (2.9 mmol) of iodomethane led to 92 mg (0.26 mmol, 89%) of isomer (*E*)-**9i**. ^1^H-NMR δ: 2.33 (4H, m); 3.64 (3H, s); 3.82 (3H, s); 3.88 (6H, s); 4.06 (2H, m); 4.61 (2H, m); 6.74 (2H, s); 6.96 (1H, d, 16.0); 7.05 (1H, d, 16.0); 7.61 (2H, d, 8.6); 7.84 (2H, d, 8.6). ^13^C-NMR δ**:** 21.2 (2) (CH_2_); 55.7 (CH_3_); 56.4 (2) (CH_3_); 61.0 (CH_3_); 66.7 (2) (CH_2_); 104.0 (2) (CH); 121.6 (2) (CH); 125.3 (CH); 128.1 (2) (CH); 131.8 (C); 132.1 (CH); 138.4 (C); 139.6 (C); 144.5 (C); 153.4 (2) (C). IR (KBr): 1583; 1247; 1125; 845 cm^−1^. Mp (acetone): 158–159 °C. HRMS: calculated for C_22_H_28_NO_3_ [M]^+^: 354.2069; found: 354.2044.

### 4.2. Aqueous Solubility Determination

Compounds were stirred in a solution of phosphate buffer at pH 7.0 (1–2 mg of compound in 300 µL of buffer) during 48 h. After that, the samples were filtered over a 45-µm filter to discard the insoluble residues. In order to determine the maximum wavelength of absorbance for each compound, a scan between 270 and 400 nm was performed. Once the wavelength was selected, a calibration line was performed and the concentration of the stirred and filtered samples was measured.

### 4.3. Cell Lines and Culture Conditions

HeLa (human cervical carcinoma) and MCF-7 (human breast carcinoma) cell lines were cultured in Dulbecco’s Modified Eagles Medium (DMEM) supplemented with 2 mM l-glutamine (Lonza-Cambrex, Karlskoga, Sweden), 10% (*v*/*v*) heat inactivated fetal bovine serum (HIFBS) (Lonza-Cambrex) and 100 μg/mL streptomycin-100 IU/mL penicillin (Lonza-Cambrex) at 37 °C in humidified 95% air and 5% CO_2_. HT-29 (human colon carcinoma) cells were cultured at 37 °C in complete RPMI 1640 medium containing 2 mM L-glutamine, 10% (*v*/*v*) HIFBS and 100 μg/mL streptomycin-100 IU/mL penicillin in humidified 95% air and 5% CO_2_ atmosphere.

### 4.4. Cell Antiproliferative Assay

The effect of the different compounds on the proliferation of human cell lines was determined by using the MTT (3-[4,5-dimethylthiazol-2-yl]-2,5-diphenyltetrazolium bromide) reagent (MTT Thiazolyl Blue Tetrazolium Bromide, Sigma-Aldrich, St. Louis, MO, USA) dissolved in PBS 5 mg/mL. Cell viability was evaluated seeding 100 µL of cells in exponential growth phase with appropriate cell line concentration (1.5 × 10^4^ HeLa and MCF-7 cells/mL and 3 × 10^4^ HT-29 cells/mL) in complete medium in 96-well plates at 37 °C and 5% CO_2_. After 24 h incubation to allow cells to attach to the plates the ligands were added at different concentrations from 10^−5^ to 10^−9^ M. The effect on the proliferation was evaluated 72 h post-treatment by adding the MTT reagent (10 µL/well) to a final concentration of 0.5 mg/mL and the plate was further incubated for another 3 h. After this period, the supernatant was removed, the MTT violet crystals formed on the bottom of each well were dissolved in DMSO (100 µL/well) and subsequently, the absorbance of the color complex was read at 570 nm using a multi-well plate reader. Compounds were dissolved in dimethyl sulfoxide (DMSO) and the final solvent concentrations never exceeded 0.5% (*v*/*v*). The control wells included treated cells with 0.5% (*v*/*v*) DMSO and the positive control. Measurements were performed in triplicate, and each experiment was repeated three times. The IC_50_ (50% inhibitory concentration with respect to the untreated controls) was determined for each compound. Nonlinear curves fitting the experimental data were carried out for each compound.

### 4.5. Cell Cycle Analysis

For the analysis of the cell cycle by flow cytometry, HeLa cells in exponential growth phase at 2 × 10^4^ cells/mL were seeded in 12-well plates (1.5 mL/well). After 24 h incubation, cells were treated with approximately 5 × IC_50_ concentration of selected compounds and the effect was measured 24, 48 and 72 h post-treatment. After the corresponding time, live and death cells were collected and fixed in ice-cold ethanol/PBS (7:3) overnight at 4 °C. Cells were harvested by centrifugation, three times-washed in phosphate-buffered saline (PBS), treated with 0.05% RNase A and stained with propidium iodide in PBS overnight at room temperature. A total of 50,000 cells were analyzed using a BD Accuri™ C6 flow cytometer and the C6 (version 1.0.264.21) software (BD Biosciences) to determine cell cycle distribution and compared to control cells.

### 4.6. Apoptotic Cell Death Assay

Apoptotic cells were quantified using an Annexin V-FITC/PI apoptosis detection kit (Immunostep, Salamanca, Spain). 1.5 mL/well of HeLa cells were seeded onto 12-well plates at a density of 2 × 10^4^ cells/mL and left to attach overnight. After 24 h cells were treated at approximately 5 × IC_50_ concentration. After 72 h and 6 days post-treatment, attached and floating cells were collected, centrifugated, resuspended in the Annexin V binding buffer and stained with Annexin V-FITC/PI according to the manufacturer’s procedure. It was incubated at room temperature for 15 min and a total of 30,000 cells were acquired using the BD Accuri™ C6 flow cytometer (BD Biosciences) as previously described.

### 4.7. Tubulin Isolation

Bovine brain microtubule protein (MTP) was isolated as previously described [23]. Briefly, it was purified according to the modified Shelanski method by two cycles of temperature-dependent assembly/disassembly [33].

### 4.8. Tubulin Polymerization Inhibition Assay

Tubulin polymerization was monitored using a Helios α spectrophotometer by measuring the increase in turbidity at 450 nm, caused by a shift from 4 °C to 37 °C, which allows the in vitro MTP self-disassembly and assembly respectively. Compounds, solved in DMSO, were prepared at the appropriate concentration in 0.1 M MES buffer, 1 mM ethylene glycol-bis(2-aminoethyl)-*N*,*N*,*N*’,*N*’-tetraacetic acid (EGTA), 1 mM MgCl_2_, 1 mM β-ME, and 1.5 mM GTP at pH 6.7 (never exceeding 4% DMSO, which has been reported not to interfere with the assembly process). Firstly, ligands at 20 µM concentration, were incubated with 1.5 mg/mL MTP at 20 °C for 30 min and subsequently cooled on ice for 10 min. Then, the experiment starts at 4 °C to establish the initial baseline. The assembly process was initiated by shifting of the temperature to 37 °C and the turbidity produced can be measured by an absorbance increase. After reaching a stable plateau, the temperature was switched back to 4 °C to return to the initial absorption values. The difference in amplitude between the stable plateau and the initial baseline of the curves was taken as the degree of tubulin polymerization for each experiment. Comparison with control curves under identical conditions but without ligands yielded tubulin polymerization inhibition as a percentage value. Finally, the IC_50_ was calculated for compounds that inhibit tubulin polymerization more than 50% at 20 µM. All the measurements were carried out in at least two independent experiments using MTP from different isolation.

### 4.9. Effect on Rat Primary Pancreatic Cells

Rat primary pancreatic cells were isolated as previously described [18]. Afterwards, pancreatic cells were resuspended in Na-HEPES solution (pH 7.4) containing 14 mM glucose, 1% essential amino acid mixture, 1% (*w*/*v*) BSA, 0.01% (*w*/*v*) STI, 0.5 mM CaCl_2_ and incubated (5% CO_2_, at 37 °C) for 7 h in the absence (non-treated control) or the presence of compounds at different concentrations. After the treatment, cells were incubated (5% CO_2_, at 37 °C) with 0.5 mg/mL MTT for 2 h and the formazan crystals were solubilized with DMSO (*v*/*v*). The optical density was read at 570 nm with a reference wavelength of 630 nm. Non-treated cell viability was set as 100%.

### 4.10. Molecular Modelling

The binding mode of compounds **1**–**9** at the colchicine site of tubulin was studied by docking experiments. The virtual ligands were built with Marvin [34]. When different isomers were possible, i.e., *Z-* and *E*- oximes (**2** and **6**) or *R*- and *S*- 1,1-diarylethanes (**4**) they were independently studied. The flexibility of the colchicine site was simulated by employing multiple configurations represented by the structures of 50 complexes of tubulin with different colchicine site ligands available at the Protein Data Bank (pdb) [35]. The protein structures were superimposed and prepared for docking with Swiss-Model [36], Chimera [37], and AutoDock Tools [38] and docking was performed with three programs employing different scoring functions: DOCK6.9 [32], PLANTS [31] and AutoDock 4.2 [38]. The docking results were semi-automatically processed by means of home-made scripts and KNIME [39] workflows that classify the binding poses according to the colchicine subsites they occupy and the combined binding energies of the three programs. The results were analyzed with AutoDockTools [38], Marvin [34], OpenEye [40], and JADOPPT [41]. Docking poses were selected if they were found by at least two docking programs using different scoring functions and based on the scoring. The selected binding mode for each ligand were those with better conjoint scoring. In order to validate the docking protocol, reference ligands with known binding modes at the colchicine site were docked using the same protocol and shown to produce the correct binding modes.

## 5. Conclusions

A new strategy for increasing molecular polarity without exposing polar groups termed masked polar group incorporation (MPGI) has been proposed as a means to increase the aqueous solubility of ligands binding at hydrophobic pockets. We applied MPGI to combretastatin analogues and designed pyridine analogues with vicinal nitrogenated substituents acting as the masking groups and synthesized a new family of compounds with a 3- or 4-pyridine ring replacing the 3-hydroxy-4-methoxyphenyl (B) ring of combretastatin A4, in an attempt to increase the aqueous solubility and remove the 3-hydroxyl group causing resistance by glucuronidation. The resulting analogues showed improved aqueous solubilities and highly potent antiproliferative activity against several cancer cell lines of different origin. The new compounds showed tubulin polymerization inhibitory activity, cell cycle arrest at the G_2_/M phase and subsequent apoptotic cell death. Docking studies suggest binding at the colchicine site of tubulin in a similar way as combretastatin A4 with the polar groups masked by the vicinal substituents. These results validate the proposed strategy for the design of colchicine site ligands and open a new road to increasing the aqueous solubility of ligands binding in apolar environments.

## Figures and Tables

**Table 1:** IC_50_ values for cell proliferation, solubility and Tubulin polymerization inhibitory (TPI) activity for the synthesized compounds.

**Compound**	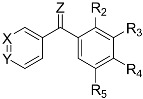							**Solub. (µg/mL)**	**HeLa IC_50_ (nM)**	**HT-29 IC_50_ (nM)**	**HT-29 IC_50_ (nM) Verap 10μM**	**MCF7 IC_50_ (nM)**	**TPI IC_50_ (μM)**
	**Z**	**Ar_1_**		**Ar_2_**									
		**X**	**Y**	**R_2_**	**R_3_**	**R_4_**	**R_5_**						
**1a**	O	CH	C-N-(CH_3_)_2_	H	OCH_3_	OCH_3_	OCH_3_	48	90	400			>20
**1b**		N	C-N-(CH_3_)_2_	H	OCH_3_	OCH_3_	OCH_3_	10.5	>1000	>1000	>1000	>1000	>20
**1c**		N	C-N-(CH_2_)_4_	H	OCH_3_	OCH_3_	OCH_3_	-	1600	5170	4000	2330	>20
**1d**		CH	C-N-(CH_3_)_2_	OCH_3_	OCH_3_	OCH_3_	H	-	413	500	650	407	>20
**1e**		CH	C-N-(CH_3_)_2_	OCH_3_	H	H	OCH_3_	-	>1000	>1000	>1000	>1000	>20
**1f**		C-N-(CH_3_)_2_	CH	H	OCH_3_	OCH_3_	OCH_3_	11	180	460	283	320	>20
**1g**		C-N-(CH_3_)_2_	N	H	OCH_3_	OCH_3_	OCH_3_	33.8	387	313	377	223	>20
**1h**		C-N-(CH_2_)_4_	N	H	OCH_3_	OCH_3_	OCH_3_	20	18	71	63	17	9.1
**2a**	NHOH	CH	C-N-(CH_3_)_2_	H	OCH_3_	OCH_3_	OCH_3_	74	800	1000			>20
**2c**		N	C-N-(CH_2_)_4_	H	OCH_3_	OCH_3_	OCH_3_	-	>1000	>1000	>1000	>1000	>20
**2d**		CH	C-N-(CH_3_)_2_	OCH_3_	OCH_3_	OCH_3_	H	-	373	320	667	277	>20
**2e**		CH	C-N-(CH_3_)_2_	OCH_3_	H	H	OCH_3_	-	>1000	>1000	>1000	>1000	>20
**2f**		C-N-(CH_3_)_2_	CH	H	OCH_3_	OCH_3_	OCH_3_	153	>1000	>1000	>1000	>1000	>20
**2g**		C-N-(CH_3_)_2_	N	H	OCH_3_	OCH_3_	OCH_3_	583	533	3370	967	407	>20
**2h**		C-N-(CH_2_)_4_	N	H	OCH_3_	OCH_3_	OCH_3_						
**3a**	CH_2_	CH	C-N-(CH_3_)_2_	H	OCH_3_	OCH_3_	OCH_3_	30	40	70			1.9
**3b**		N	C-N-(CH_3_)_2_	H	OCH_3_	OCH_3_	OCH_3_	13.5	150	277	100	81	35.9
**3c**		N	C-N-(CH_2_)_4_	H	OCH_3_	OCH_3_	OCH_3_	-	523	383	140	-	>20
**3d**		CH	C-N-(CH_3_)_2_	OCH_3_	OCH_3_	OCH_3_	H	-	1000	4000	1500	2000	>20
**3e**		CH	C-N-(CH_3_)_2_	OCH_3_	H	H	OCH_3_	-	>1000	>1000	>1000	>1000	>20
**3f**		C-N-(CH_3_)_2_	CH	H	OCH_3_	OCH_3_	OCH_3_	21	>1000	>1000	>1000	>1000	>20
**3g**		C-N-(CH_3_)_2_	N	H	OCH_3_	OCH_3_	OCH_3_	136	403	397	380	367	>20
**3h**		C-N-(CH_2_)_4_	N	H	OCH_3_	OCH_3_	OCH_3_	62	257	477	230	317	>20
**4d**	CH_3_	CH	C-N-(CH_3_)_2_	OCH_3_	OCH_3_	OCH_3_	H	-	>1000	>1000	>1000	>1000	>20
**4e**		CH	C-N-(CH_3_)_2_	OCH_3_	H	H	OCH_3_	-	>1000	>1000	>1000	>1000	>20
**4f**		C-N-(CH_3_)_2_	CH	H	OCH_3_	OCH_3_	OCH_3_	-	>1000	>1000	>1000	>1000	>20
**5a**	O	CH	C-N^+^-(CH_3_)_3_	H	OCH_3_	OCH_3_	OCH_3_	-	>1000	>1000	>1000	>1000	>20
**5d**		CH	C-N^+^-(CH_3_)_3_	OCH_3_	OCH_3_	OCH_3_	H	4400	>1000	>1000	>1000	>1000	>20
**5e**		CH	C-N^+^-(CH_3_)_3_	OCH_3_	H	H	OCH_3_	>5000	>1000	>1000	>1000	>1000	>20
**6a**	NHOH	CH	C-N^+^-(CH_3_)_3_	H	OCH_3_	OCH_3_	OCH_3_	1500	>1000	>1000	>1000	>1000	>20
**7a**	CH_2_	CH	C-N^+^-(CH_3_)_3_	H	OCH_3_	OCH_3_	OCH_3_	2500	>1000	>1000	>1000	>1000	>20
**7d**		CH	C-N^+^-(CH_3_)_3_	OCH_3_	OCH_3_	OCH_3_	H	1000	>1000	>1000	>1000	>1000	>20
**Compound**	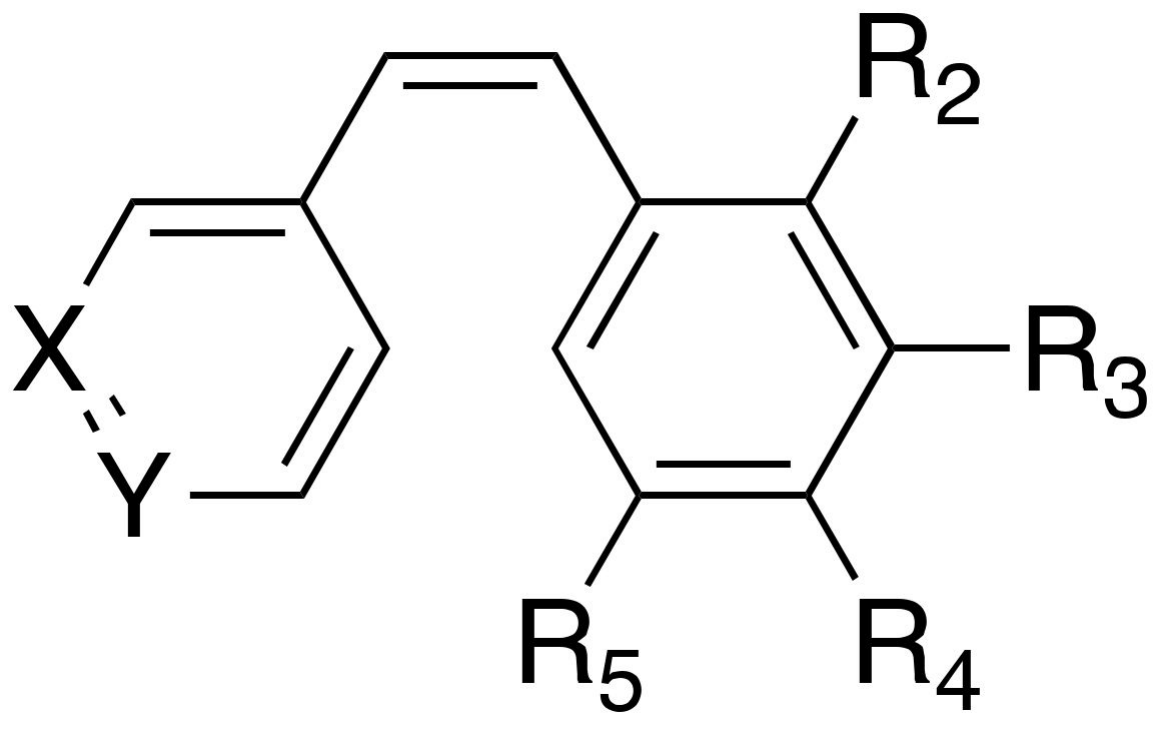							**Solub. (µg/mL)**	**HeLa IC_50_ (nM)**	**HT-29 IC_50_ (nM)**	**HT-29 IC_50_ (nM) Verap 10μM**	**MCF7 IC_50_ (nM)**	**TPI IC_50_ (μM)**
	**Ar_1_**			**Ar_2_**									
	**X**		**Y**	**R_2_**	**R_3_**	**R_4_**	**R_5_**						
**8a**	CH		C-N-(CH_3_)_2_	H	OCH_3_	OCH_3_	OCH_3_	-	208	503	102	273	3.4
**8b**	N		C-N-(CH_3_)_2_	H	OCH_3_	OCH_3_	OCH_3_	44	57	78	50	63	5.1
**8c**	N		C-N-(CH_2_)_4_	H	OCH_3_	OCH_3_	OCH_3_	-	>1000	>1000	>1000	>1000	>20
**8h**	C-N-(CH_2_)_4_		N	H	OCH_3_	OCH_3_	OCH_3_	65	>1000	>1000	>1000	>1000	>20
**8i**	CH		C-N-(CH_2_)_4_	H	OCH_3_	OCH_3_	OCH_3_	-	243	430	73	140	-
**9a**	CH		C-N^+^-(CH_3_)_3_	H	OCH_3_	OCH_3_	OCH_3_	-	>1000	>1000	>1000	>1000	>20
**9b**	N		C-N^+^-(CH_3_)_3_	H	OCH_3_	OCH_3_	OCH_3_	679	>1000	>1000	>1000	>1000	>20
**9i**	CH		C-N^+^-(CH_3_)-(CH_2_)_4_	H	OCH_3_	OCH_3_	OCH_3_	-	>1000	>1000	>1000	>1000	-

**Table 2:** Toxicity against rat primary pancreatic cells. Results are expressed as a percentage (means ± S.E.M.) of live cells with respect to the non-treated control, taken as 100%.

1h		1f		3b		8b		NaTc	Doxorubicin	Acetyldigoxin	Etoposide
0.50 µM	0.25 µM	0.50 µM	0.25 µM	0.50 µM	0.25 µM	0.50 µM	0.25 µM	0.50%	0.05 µM	0.05 µM	0.05 µM
102.59 ± 14.73	103.84 ± 9.20	95.97 ± 10.55	106.59 ± 17.60	94.06 ± 13.13	112.81 ± 19.30	91.65 ± 13.88	94.31 ± 11.83	24.83 ± 11.86	53.09 ± 10.27	84.41 ± 9.64	89.87 ± 11.80

## Supplementary Materials

The following are available online: Table S1: the details of the docking results.
