# Supplementary material for: The Masked Polar Group Incorporation (MPGI) Strategy in Drug Design: Effects of Nitrogen Substitutions on Combretastatin and Isocombretastatin Tubulin Inhibitors
Source: Molecules. 2019 Nov 26;24(23):4319. doi: 10.3390/molecules24234319 (PMC6930638; doi:10.3390/molecules24234319)
Supplement: Supplementary file 1 [file molecules-24-04319-s001.pdf]

Supplementary Material:

Table S1. Docking Results

Comp.	Cytotoxicity range (nM)[a]	SUBS[b]	PROT[c]	Zscore[d]	PLANTS[e]	AD4[f]	AMBfix[g]	AMBmov[h]
**1b**	>1000	AB	5H7O	0,81	-68,22		-47,81	-47,79
		AB	5JVD	0,81		-7,67	-41,70	-51,35
**1c**	>1000	AB	5H7O	0,69	-76,69		-47,66	-45,23
		BC	CA4	0,69		-8,67	-40,32	-33,62
**1d**	407-650	AB	5Z4P	0,78	-61,53		-44,52	-58,33
		AB	CA4	0,78		-7,47	-46,72	-44,25
**1e**	>1000	AB	5H7O	0,72	-84,09		-52,34	-52,47
		AB	5JVD	0,72		-7,77	-39,24	-44,27
**1f**	180-460	AB	5Z4P	0,92	-66,99		-35,18	-57,30
		AB	6D88	0,83		-7,73	-37,62	-48,46
**1g**	223-387	AB	6BRY	0,85		-7,39	-30,51	-26,14
		AB	5NFZ	0,85	-62,73		-27,51	-51,72
**1h**	17-71	ABC	5Z4P	0,72	-76,89		-38,26	-48,56
		BC	5JVD	0,72		-9,08	-43,97	-58,04
***E*-2c**	>1000	BC	5JVD	0,73		-9,80	-39,42	-52,93
		AB	5H7O	0,73	-66,10		-43,25	-44,41
***Z*-2c**	>1	BC	5JVD	0,65		-9,93	-44,34	-48,96
		AB	5H7O	0,65	-79,50		-42,94	-46,97
***E*-2d**	277-677	AB	5Z4P	0,77	-54,69		-45,82	-51,67
		AB	5XKH	0,76		-8,46	-38,24	-42,30
***Z*-2d**	277-677	AB	5Z4P	0,77	-54,39		-48,67	-40,22
		AB	CA4	0,77		-8,12	-44,40	-49,81
***E*-2e**	>1000	AB	5JVD	0,75	-78,66		-46,89	-44,95
		AB	5H7O	0,75		-8,24	-52,00	-51,20
***Z*-2e**	>1000	AB	5JVD	0,78		-8,42	-34,79	-38,30
		AB	5H7O	0,78	-81,95		-54,10	-53,76
***E*-2f**	>1000	AB	5NFZ	0,89	-63,94		-31,04	-42,53

		AB	5JVD	0,88		-8,25	-34,03	-45,13
***Z*-2f**	>1000	AB	6F7C	0,87	-64,08		-17,66	-25,63
		AB	5JVD	0,87		-8,49	-39,24	-40,05
***E*-2g**	407-3330	AB	5XAF	0,83	-61,63			
		AB	CA4	0,83		-8,03	-34,60	-35,89
***Z*-2g**	407-3330	AB	5H7O	0,82	-62,57			
		AB	6BRY	0,82		-7,81	-25,44	-27,14
**3b**	81-277	AB	5H7O	0,83	-73,49		-47,36	-57,42
		AB	5JVD	0,83		-8,13	-47,39	-43,99
**3c**	140-523	BC	5JVD	0,68		-9,58	-45,78	-53,54
		AB	5H7O	0,68	-79,64		-9,57	-47,69
**3d**	>1000	AB	5Z4P	0,76	-66,49		-57,21	-47,12
		AB	5H7O	0,76		-8,09	-50,95	-38,49
**3e**	>1000	AB	5H7O	0,75	-86,54		-52,83	-59,52
		AB	5H7O	0,75		-7,93	-56,12	-53,42
**3f**	>1000	AB	5Z4P	0,90	-73,45		-41,62	-45,56
		AB	6BRY	0,90		-7,97	-35,02	-44,20
**3h**	230-477	ABC	5Z4P	0,69	-79,92		-40,62	-46,71
		AB	CA4	0,69		-9,33	-41,32	-45,58
**4d**	>1000	AB	5Z4P	0,82	-67,36		-44,88	-48,65
		AB	6BRY	0,81		-7,86	-36,79	-47,23
**4d**	>1000	AB	6F7C	0,65	-60,38		-44,91	-51,66
		AB	6BRY	0,65		-7,88	-42,43	-49,63
**4e**	>1000	AB	5JVD	0,78	-88,48		-43,83	-53,96
		AB	5JVD	0,78		-8,19	-44,46	-44,70
**4e**	>1000	AB	5JVD	0,79		-7,97	-43,66	-54,27
		AB	5Z4P	0,79	-91,66		-50,46	-61,24
**4f**	>1000	AB	5Z4P	0,96	-69,96		-35,32	-45,85
		AB	CA4	0,95		-7,77	-39,61	-51,91
**4f**	>1000	AB	5Z4P	0,95	-72,55		-33,32	-37,16

		AB	CA4	0,94		-8,71	-42,27	-61,37
**4g**	367-403	AB	5Z4P	0,82	-70,78		-30,87	-39,74
		AB	5JVD	0,82		-7,68	-38,38	-39,82
**5a**	>1000	AB	5JVD	0,74		-8,73	-11,61	-30,41
		AB	bLact	0,74	-56,46		-21,14	-27,39
**5d**	>1000	AB	5H7O	0,70	-53,67		-25,27	-46,25
		AB	CA4	0,70		-8,10	-12,72	-41,90
**5e**	>1000	AB	5H7O	0,67	-75,84		-23,40	-32,14
		AB	5H7O	0,67		-8,28	-19,64	-31,91
***E*-6a**	>1000	AB	CA4	0,75	-59,03		-14,52	-40,75
		AB	5JVD	0,75		-9,31	-14,95	-32,97
***Z*-6a**	>1000	AB	5H7O	0,72	-61,58		-27,84	-32,08
		AB	5JVD	0,72		-9,34	-18,37	-36,40
**7a**	>1000	AB	5H7O	0,73	-66,85		-34,98	-47,30
		AB	5JVD	0,73		-9,17	-22,57	-43,51
**7d**	>1	AB	5Z4P	0,70	-58,48			
		AB	5H7O	0,70		-8,89	-30,71	-51,38
**8a**	102-503	AB	CA4	0,82	-76,61		66,82	-27,56
		AB	CA4	0,82		-8,66	-56,37	-51,33
**8b**	57-80	AB	5H7O	0,89	-67,33		-48,23	-54,11
		AB	CA4	0,89		-7,87	-42,17	-41,51
**8c**	>1000	ABC	5JVD	0,70	-79,79		-32,56	-55,76
		BC	5JVD	0,70		-10,07	-50,83	-56,07
**8h**	>1000	AB	5JVD	0,72		-9,31	-31,21	-43,32
		AB	5H7O	0,72	-76,49		-33,76	-45,46
**9a**	>1000	AB	5H7O	0,77	-64,56		-31,09	-38,73
		AB	CA4	0,77		-9,03	-25,06	-47,86

Supplementary Table 1. Docking results. [a] Range of antiproliferative IC_{50} values. [b] Subsites of the colchicine site occupied by the selected pose. [c] PDB-ID with the best docking score for the selected pose. [d] Z score of the selected pose within the Autodock or PLANTS scores. [e] PLANTS score for the selected pose. [f] AutoDock4 score for the selected pose. [g] MM-PBSA with AMBER for

the selected pose after 5 ns molecular dynamics with the protein fixed. [b] MM-PBSA with AMBER for the selected pose after 5 ns molecular dynamics with the protein free.
